# Application of the Malaria Management Model to the Analysis of Costs and Benefits of DDT versus Non-DDT Malaria Control

**DOI:** 10.1371/journal.pone.0027771

**Published:** 2011-11-30

**Authors:** Matteo Pedercini, Santiago Movilla Blanco, Birgit Kopainsky

**Affiliations:** 1 Millennium Institute, Washington, District of Columbia, United States of America; 2 System Dynamics Group, Department of Geography, University of Bergen, Bergen, Norway; 3 Flury&Giuliani GmbH, Zurich, Switzerland; Burnet Institute, Australia

## Abstract

**Introduction:**

DDT is considered to be the most cost-effective insecticide for combating malaria. However, it is also the most environmentally persistent and can pose risks to human health when sprayed indoors. Therefore, the use of DDT for vector control remains controversial.

**Methods:**

In this paper we develop a computer-based simulation model to assess some of the costs and benefits of the continued use of DDT for Indoor Residual Spraying (IRS) versus its rapid phase out. We apply the prototype model to the aggregated sub Saharan African region. For putting the question about the continued use of DDT for IRS versus its rapid phase out into perspective we calculate the same costs and benefits for alternative combinations of integrated vector management interventions.

**Results:**

Our simulation results confirm that the current mix of integrated vector management interventions with DDT as the main insecticide is cheaper than the same mix with alternative insecticides when only direct costs are considered. However, combinations with a stronger focus on insecticide-treated bed nets and environmental management show higher levels of cost-effectiveness than interventions with a focus on IRS. Thus, this focus would also allow phasing out DDT in a cost-effective manner. Although a rapid phase out of DDT for IRS is the most expensive of the tested intervention combinations it can have important economic benefits in addition to health and environmental impacts that are difficult to assess in monetary terms. Those economic benefits captured by the model include the avoided risk of losses in agricultural exports.

**Conclusions:**

The prototype simulation model illustrates how a computer-based scenario analysis tool can inform debates on malaria control policies in general and on the continued use of DDT for IRS versus its rapid phase out in specific. Simulation models create systematic mechanisms for analyzing alternative interventions and making informed trade offs.

## Introduction

Malaria is one of the world's most deadly diseases, and it is especially dangerous for children and pregnant women. Every year around 780'000 people die from malaria and more than 225 million cases of clinical malaria are reported [1]. Around 90% of these cases happen in sub Saharan Africa (SSA) [2]. In addition to the death toll, malaria has strong implications for development. Malaria has a relevant impact on workers' productivity, thus reducing a country's economic growth prospects. Malaria also absorbs a large amount of funds that could otherwise be used for investment in productive activities. In addition, malaria reduces students' attendance at school, thus affecting their education and productivity in the long run. Therefore, countries where malaria is endemic are often stuck in a malaria trap, where malaria is at the same time a cause for and an effect of slow development [3].

Malaria interventions focus on both, case management (treatment) and prevention [4]. Successful case management is based on prompt disease recognition and on the use of adequate and high-quality therapies for eradication of the parasite species causing malaria. On the other hand, methods for prevention are very diverse. Excluding blood transfusion and congenital transmission, Anopheline mosquito species are the only known malaria vector. Almost all preventive measures are thus intended to avoiding mosquito bites to humans. Vector control measures include environmental, mechanical, biological, and chemical as well as structural adaptations that can be allocated to two broad categories [4]: Indoor Residual Spraying (IRS) and Insecticide Treated Bed nets (ITN). In this paper we focus on vector control and thus on malaria prevention interventions. In addition to IRS and ITN for vector control, we also include Environmental Management (EM) because it has yielded promising results in certain environments and becomes increasingly more important as vectors develop resistance to insecticides and parasites to drugs [5], [6], [7].

As no intervention in isolation is able to control malaria transmission and as mosquitoes adapt to some interventions, integration and coordination of various prevention interventions is essential to achieve malaria elimination. Integrated Vector Management (IVM) should provide intelligent management of prevention interventions, the optimal use of existing resources to fight malaria, advocacy and social mobilization as well as capacity building [8].

Assessing the costs and effectiveness of different combinations of IVM interventions requires a comprehensive understanding of the many indirect, delayed, and nonlinear feedback effects that IVM interventions can have. Building such understanding can be supported considerably by experimentation with computer simulation models that allow the design and analysis of different scenarios and policies in a risk free environment [9]. This paper develops a simulation based Malaria Management Model (MMM). The MMM contains a representation of malaria transmission processes, vector control, and case management, and it dynamically links these components to population development, health, education, and economic production.

A first pilot version calibrates and applies the model to the aggregated sub Saharan African region and assesses the costs and benefits of the continued use of DDT for IRS. One fundamental and often controversial category of vector control interventions is Indoor Residual Spraying (IRS). IRS is a predominantly chemical vector control designed to eliminate or repel mosquitoes. This method with its protective features against malaria transmission merits special attention because vectors can develop resistances to the sprayed chemicals and because the sprayed chemicals can be a threat for both human health and the environment [10], [11]. One of the twelve World Health Organization-(WHO) approved insecticides for IRS is dichloro-diphenyl-trichloroethane (DDT). According to WHO [12], DDT is the most cost-effective and longest-lasting insecticide but at the same time also the most environmentally persistent [13]. When sprayed indoors, it can pose risks to human health [14]. Therefore, the use of DDT for vector control remains controversial. The Stockholm Convention on Persistent Organic Pollutants aims at a complete phase out of DDT but currently allows it for disease vector control when locally safe, effective, and affordable alternatives are not available.

With the Malaria Management Model, we calculate the costs of eliminating malaria either with the currently used mix of IVM interventions with DDT as the most prominent insecticide or with alternative insecticides. With malaria elimination we refer to the interruption of transmission, which requires continuous measures to prevent re-establishment of transmission. Malaria eradication, on the other hand, would imply the permanent reduction to zero of the worldwide incidence of infection where intervention measures are no longer needed (e.g., [4]).

For putting the question about the continued use of DDT for IRS versus its rapid phase out into perspective, we calculate the same costs and benefits for alternative combinations of IVM interventions, i.e., we calculate the costs of eliminating malaria in sub Saharan Africa and how these costs compare to the gains in GDP that would result from malaria elimination for each combination. We perform these calculations for an ambitious short-term time horizon (elimination by 2025) and a mid-term time horizon (elimination by 2035). With this pilot application of the MMM we thus address the following research questions:

1. What is the amount of resources necessary to gradually scale up the current combination of vector control interventions for achieving malaria elimination in 2025 or in 2035, respectively?
2. How does this amount compare to the gain in GDP that could be achieved through malaria elimination?
3. What are the costs and benefits of the continued use of DDT for IRS versus its rapid phase out? The indirect or external costs of DDT, i.e., the costs related to the environmental and health impacts, are difficult to assess in monetary terms, and they also depend on value judgments and risk attitudes [15]. For this reason we only calculate the direct costs such as the price per DDT-intervention and the direct benefits such as impacts on gross domestic production (GDP). We add some indications of external costs and risks to highlight how they can affect evaluations of the effectiveness of the continued use of DDT for IRS versus its rapid phase out.
4. What is the amount of resources necessary to gradually scale up alternative combinations of vector control interventions for achieving malaria elimination in 2025 or 2035, respectively?
5. How do these amounts compare to the gain in GDP that could be achieved through malaria elimination?

An effective IVM strategy needs to be designed based on location-specific ecological and epidemiological characteristics and social factors [5], [15]. It is thus very difficult to determine the optimal combination of interventions for scaling up vector control to achieve malaria elimination in the SSA region. Sub Saharan Africa is a broad and diverse region with varied eco-epidemiological situations. In this study we do not aim at identifying a specific combination of interventions that would fit all situations. Instead, we recognize that different interventions fit different situations. Based on this consideration, we analyze the likely necessary budget to eliminate malaria for the aggregate SSA region under different assumptions regarding what type of interventions might be most needed without assuming that all interventions will be identical in all locations. We then compare these costs to the likely economic benefits of malaria elimination (cost-effectiveness of different combinations of vector control interventions). Finally, for our pilot application of the MMM to the question about the continued use of DDT for IRS, the aggregate SSA region provides a sufficiently homogenous level of analysis. Further applications of the MMM, however, will require more location-specific analysis, e.g., country-level analyses.

The prototype Malaria Management Model illustrates how a computer based scenario analysis tool can inform debates on malaria control policies in general and on the continued use of DDT for IRS versus its rapid phase out in specific. The model separates issues of scientific uncertainty such as the impact of DDT for IRS on human health and the environment from disagreement over values, i.e., over the weight that should be put on longer term human health, economic and environmental impacts versus short term reductions in direct costs when DDT is used for IRS. Simulation models cannot resolve such disagreements. However, they can highlight the role of the different aspects related to the design of IVM interventions and provide a user-friendly tool that allows decision makers to explore the impact of different component weights on the costs and benefits of such interventions. Simulation models create more systematic mechanisms for analyzing alternative interventions and making informed trade offs.

## Materials and Methods

This paper adopts a system dynamics approach. System dynamics is a computer aided approach to policy analysis and design. It applies to dynamic problems (problems that involve change over time) arising in complex social, economic, or ecological systems, i.e., in systems characterized by feedback loops, delays, and nonlinearities [16], [17]. An example for a feedback loop in the context of this paper is the circular causality between economic production and malaria where increases in economic production enable higher malaria expenditures, which help reducing malaria prevalence. This, in turn has a beneficial effect on economic production as the labor force becomes more productive and thus increases economic production even further. Delays are omnipresent, also in this example of a feedback loop, as time elapses between increases in malaria expenditures and observable increases in labor force productivity. Examples for nonlinearities are the elasticities with which total factor productivity (an element of the economic production function) changes in accordance with changes in the education or health levels of the population.

The system dynamics approach involves the development of a simulation model. Experimentation with computer simulation models allows the design and analysis of different scenarios and policies in a risk free environment [9]. Dynamic simulation models are sets of equations that describe the behavior of dynamic systems. The models study cause and effect. Given specified initial conditions and assumed behavioral parameters, the models trace the changes in key variables over time and allow seeing the dynamic implications of the assumptions [18]. Mathematically, the basic structure of a formal dynamic simulation model consists of a system of coupled, nonlinear, first-order differential (or integral) equations. Simulation of such systems is accomplished by partitioning simulated time into discrete time intervals of length *dt* and stepping the system through time one *dt* at a time. By breaking the simulated time into discrete intervals *dt* simulation makes possible the creation and use of models that cannot be solved in closed form. Simulation thus expands the range and complexity of problems that can be modeled [16].

### Structure of the simulation model

The Malaria Management Model contains an aggregated representation of the malaria transmission process, integrated vector management with a special focus on DDT, and malaria case management (diagnosis and treatment) in sub Saharan Africa. This malaria sector of the model is based on research carried out in the context of the Community Level Model project [19] and it is integrated into a broader socio-economic development framework that traces the most important social and economic aspects of development in the SSA region. This framework is based on the Threshold21 (T21) simulation model structure developed by the Millennium Institute [20]. T21 provides an integrated representation of the fundamental socio-economic and environmental development mechanisms [20]. It is a scenario analysis tool designed to support national development planning and has been successfully applied in over 25 countries (e.g., [21], [22]). In this pilot stage, we apply the simulation model to the aggregate sub Saharan African region (SSA), where the vast majority of malaria infections occur [2]. The boundaries of SSA provide a reasonably homogenous malaria region for the generic analysis of costs and benefits of the continued use of DDT for IRS.

The resulting Malaria Management Model (MMM) keeps the broad and integrated approach that characterizes the T21 framework. At the same time, it is specifically developed with an emphasis on representing and analyzing the dynamics of the malaria problem. The model thus supports an integrated assessment of malaria control policies and their long-term development implications. Figure S1 provides an aggregated representation of the structure of the MMM. The structure is composed of five sectors: population, production, education, health, and malaria. All sectors interact with each other and the arrows between the sectors Figure S1 describe the directions of these interactions.

The *population sector* calculates the total population in the SSA region based on births, deaths and migration [23], [24]. For the calculations, population is divided into one-year age cohorts and differentiated by gender. Fertility, and thus the number of births, depends on income and education [25]. Mortality, and thus the number of deaths, is determined by life expectancy (which is calculated in the health sector) and malaria. Under-five mortality is used as an input to calculate fertility, so that with higher under-five mortality rate (of which malaria deaths are an important component) fertility rate is also higher. This is an important development mechanism that tends to balance the increase in population due to lower mortality with a decrease in fertility.

The *education sector* describes the process of acquiring education and thus becoming literate through public and private systems. Access to education and thus eventually the average adult literacy rate depends on the expenditure for education.

The *health sector* reproduces how access to health care and thus life expectancy change over time depending on the level of income and on the expenditure for health care [26], [27], [28]. The impact of malaria on deaths is calculated in the population sector.

The *production sector* calculates the economic production of goods and services by using an extended Cobb-Douglas production function. Due to its simplicity and flexibility, the extended Cobb-Douglas production function has been extensively used for long-term development analysis in a variety of developing countries [21], [29], [22]. It is especially well suited for our application, in which we focus on long-term economic trends, where we only need a low level of detail with respect to the process of economic production. Total factor productivity (TFP) is determined based on the overall levels of education and health. The effect of malaria prevalence on productivity is also taken into account. We do not explicitly consider distributional issues, that is, we use average figures (e.g., average income, average level of education, average life expectancy) as elements affecting economic, social, and epidemiological trends. In reality, the poorest, least educated, and physically weaker individuals and families are the most affected by malaria. By working with average figures, we do not neglect that those poorest households are the most affected by malaria, but we assume that inequality of distribution will not fundamentally change over the time horizon of the simulation. Since our policy analysis is not concerned with distributional issues and redistribution policies, this assumption seems consistent with the scope of our work. Nevertheless, the inclusion of inequality dynamics in the analysis of malaria diffusion is a fascinating area for further research.

The *malaria sector* occupies a central role in the framework since it fundamentally affects development in the other sectors and is at the same time also affected by the development in the other sectors (Figure S1). The malaria sector itself is split into five subsectors (Figure S2): malaria transmission, IVM interventions, case management, cost accounting, and DDT concentrations, which are described in more detail below.

- Malaria transmission: This subsector calculates the number of malaria deaths per year. This calculation is based on the size of the malaria infectious population and on malaria mortality, which depends on the efficacy and coverage of case management. The malaria infectious population results from the vulnerable population and the malaria infection rate, i.e., the rate at which the vulnerable population is infected with malaria. The vulnerable population is determined based on the estimated proportion of the population living in risk areas, and on the effective coverage of malaria prevention, i.e., of IVM interventions. Climatic conditions play an important role for malaria transmission: more suitable climate conditions may facilitate malaria transmission. The extension of the malaria risk areas, and thus the vulnerable population, depends on climatic conditions such as temperature, rainfall and humidity. We use the Malaria Transmission Climate Suitability Index (MTCSI, e.g., [30], which defines the level of risk for six disaggregated sub regions in SSA, to determine the vulnerable population. The model does not explicitly represent the parasite cycle in the vector since these are rapid processes (in the order of a few weeks) whose dynamics would not be relevant for long-term simulation. Instead, the model represents the acquisition and persistence of the parasite in the human body, which is a fundamental process driving the diffusion of the disease.
- IVM interventions: This subsector represents the implementation mechanisms and related costs of selected IVM interventions. IVM interventions include various protective measures which we summarize into insecticide-treated bed nets (ITN), indoor residual spraying (IRS), and environmental management (EM). To determine IVM coverage, we consider the cumulative units of intervention deployed and their depreciation over time (which is quite long, for example, for bed nets, while quite short for IRS treatment). We consider all IVM interventions and their individual effectiveness, and then use an estimated 50% overlapping factor among IVM interventions to account for the fact that no intervention alone can ultimately eliminate malaria (e.g., [31]). Possible developments regarding vaccination are not considered, as the time required for developing such vaccination, its potential effectiveness, and the resources involved remain highly uncertain. The *use of ITN* has turned out to be very effective, especially when bed nets are properly used. The big advantage of this protective measure is that the bed nets are relatively cheap and that they guarantee protection for three or four years if properly maintained. However, if misused, these benefits are almost entirely canceled. In the MMM model this is reflected by the average education level which determines the effectiveness of ITN. *IRS* is a predominantly chemical vector control method consisting of the indoor spraying of insecticides to kill or repel mosquitoes. The different insecticides used for IRS have different unit costs and different residual times on walls. We integrate the average annual costs per person into the model by calculating the unit costs of the current mix of insecticides and the costs of a mix with no DDT (Table S1; Table 1). The costs covered by the MMM include all costs associated with spray operations, management and administration, and technical assistance [32]. One of the significant limiting factors of IRS is that it is labor intensive and that mosquitoes develop resistances [11]. The MMM model considers different resistance factors for the different insecticides [11]. *Environmental management* consists of environmental manipulation (e.g., periodic removal of aquatic weeds or riverine vegetation, alternating cycles of irrigation and dry farming), environmental modification (e.g., capital-intensive investments that lead to permanent changes such as landscaping, drainage, land reclamation and filling), and strategies that reduce contacts between mosquitoes and humans (e.g., house screening) [33], [34], [35]. We also include larviciding, i.e., the direct application of larval control agents to larval habitats for killing the mosquito larvae, in the environmental management subsector [6], [7].
- Case management: This subsector keeps track of treatment coverage and costs for the malaria infected population. Treatment coverage depends on specific malaria treatment expenditure, but also on generic health expenditure per capita, since this determines coverage of basic health services. The effective treatment coverage is determined by the average efficacy of the malaria treatments (which can be reduced by increasing drug resistance) and by the percentage of infected people who attend formal health services. This percentage increases with improvements in the general education level. Effective treatment coverage is a good indicator to estimate malaria mortality among the infected population. The malaria transmission, IVM interventions, and the case management sectors are visualized in Figure S2.
- DDT concentrations: This sub-sector represents the process of DDT production-distribution-use-dispersion at the global scale. It keeps track of the production and use of DDT for agriculture and malaria control. Global volumes of trade and concentrations of DDT in the environment are also represented in this sector. Tracking DDT concentrations in soil, air, oceans or fish allows the assessment of possible DDT impacts on human health and the environment.
- Cost accounting: The cost accounting subsector summarizes the economic costs of malaria (effect of malaria on productivity) and of the implemented prevention and case management interventions (case management and prevention expenditures) depicted in the simulation model. It also calculates the long-term impacts of malaria on human health (effect of malaria on life expectancy, effect of DDT on life expectancy, fraction of the population affected by malaria) and the environment (DDT concentrations). Additional costs such as monitoring of human health and environmental exposure levels, repatriation of waste, repatriation of unused stocks or safe disposal of unused stocks of DDT are not included in our cost accounting.

**Table 1:** Assumptions used for the mix of IVM interventions in the policies.

	Current policy mix	Current policy mix without DDT	Policy mix focusing on ITN	Policy mix focusing on IRS	Policy mix focusing on EM
ITN	55%	49.5%	66%	33.5%	55%
IRS	44.5% of which 85% DDT	50%	33.5% of which 85% DDT	66% of which 85% DDT	35% of which 85% DDT
EM	0.5%	0.5%	0.5%	0.5%	10%
Research question addressed	1: amount of resources necessary to achieve malaria elimination with current mix of IVM interventions; 2: costs compared to gains in GDP	3: costs and benefits associated with continued use of DDT vs. its rapid phase out	4: amount of resources necessary to achieve malaria elimination with alternative mixes of IVM interventions; 5: costs compared to gains in GDP		

Notes:

- The current policy mix is based on the costs per person covered listed in Table S1, on [48] for total quantities of each insecticide used for IRS, on [4] for ITN coverage and on Table S1 for EM coverage and resulting percentage in policy mix.

- The percentages in each intervention category in the current policy mix without DDT differs from the current policy mix scenario because we assume the same degree of coverage with IRS than in the current mix scenario. As the direct costs for non-DDT chemicals are higher than the direct costs of DDT, more money needs to be allocated to IRS so that less can be allocated to ITN and EM (not visible in the case of EM due to rounding effects).

### Data and assumptions

The MMM model is long-term in scope and covers the historical period between 1970 and 2010 as well as projections into the future until the year 2050. The long historical period provides a means for calibrating and validating the simulation model while the long projection period into the future allows a long-term perspective on malaria and socio-economic dynamics.

The simulation model is applied to the aggregate SSA region. This global scope allows estimating the budget requirements for eliminating malaria under different assumptions concerning the relevant time horizon and the mix of vector control interventions.

An important step in the model-building process is to specify mathematical equations for each of the relationships in the model and to quantify the model's parameters. One set of parameters are the initial values for the stocks. Another set are the constants in the model. Data is also used for the calibration of the simulation model. In this case, the time series for a particular variable which is produced by the model is compared to a time series perceived in the system. Parameters with no available data source can be estimated or calibrated such that the simulated time series for other variables matches the observed data for that variable as closely as possible. Table S1 provides an overview of the data used for the specification and calibration of malaria sectors in the MMM model. Data used in the model is based on available statistical data and on relevant literature (values and references indicated in Table S1). The structure of the simulation model was validated in a series of expert interviews. The most prominent data source for the population, education, health and production sectors are the United Nations Population Division, The World Bank's Education Statistics Database, and The World Bank's World Development Indicators. The technical appendix Text S1 lists all the equations, initial values and parameter values of the simulation model. The simulation model itself is available as online supporting information (Dataset S1 and Dataset S2).

### Policies and scenarios

For testing different policies and scenarios, a baseline has to be established. The baseline scenario reproduces historical behavior as closely as possible. For the time period in the future it assumes no fundamental shifts in malaria policies, and no major external shocks. More specifically, regarding malaria policies, we assume that malaria expenditure (measured as its share on the total gross domestic product GDP in the SSA region) grows by 1 percent per year. Health and education expenditures remain a fixed share of GDP.

After establishment of the baseline simulations, the MMM model can be used to address the research questions formulated in the introduction section of this paper. The key question underlying this paper concerns the costs and benefits of a continued use of DDT for IRS versus its rapid phase out. For comparison purposes, we calculate the same costs and benefits for alternative combinations of IVM interventions. For all calculations we consider the costs necessary for eliminating malaria as well as the benefits resulting from malaria elimination. Costs and benefits are calculated on the basis of a 3% discount rate.

In the MMM model, malaria elimination becomes possible if and when the entire vulnerable population is effectively covered by IVM interventions. The model calculates the coverage of each IVM intervention resulting from the expenditures for this intervention. To achieve effective coverage of the entire vulnerable population, the model assumes an overlapping factor of 50% between the different IVM interventions.

In addition to calculating the costs of elimination, we compare these costs to the gain in GDP that could be achieved through malaria elimination. Focusing on the gain in GDP integrates a series of other benefits gained from malaria elimination such as saved lives. Saved lives, in the MMM model, translate into a healthier and more productive workforce that increases the overall level of GDP.

After calculating the baseline as well as the economic costs of malaria (potential gain in GDP), we define a series of policies that we test with the MMM model and that we compare to the baseline. The policies differ in their combination of IVM interventions (Table 1). The policies are subjected to two different scenarios. The first scenario aims at eliminating malaria in SSA by 2025, the second by 2035. Table 1 details the assumptions underlying the different policies, i.e., the different combinations of IVM interventions and specifications of Indoor Residual Spraying (IRS), Insecticide Treated Bed Nets (ITN), and Environmental Management (EM). The table also lists the research questions that can be answered with such analyses. The percentages of the interventions in the table relate to the percentage of the corresponding intervention category on total malaria prevention expenditure. The choice of policies is based on two criteria. First, we define policies such that they test combinations of IVM interventions with different emphases. Second, the combinations need to be a realistic possibility of allocating the different IVM interventions to the aggregated SSA region. The results obtained from these policies should give a sense of the magnitude and direction of the change in costs necessary for eliminating malaria. Further emphasis in one direction in the mix will cause even more changes in the costs (in the same direction).

For assessing the costs and benefits of a policy-scenario combination, we calculate the malaria expenditures necessary to gradually scale up the specific combination of IVM interventions for achieving malaria elimination in 2025/35. We implement expenditure policies by setting different levels of malaria expenditure as fixed shares of SSA GDP. This implies that we assume stable financial support from development partners (77% of total malaria expenditure). This assumption might not be realistic as international financial support is likely to fluctuate. However, it demonstrates the need for stable financial support over extended periods of time if malaria elimination is to be reached at all.

The current mix of interventions in terms of malaria expenditure consists of 55% ITN, 44.5% IRS and 0.5% EM (Table 1). For scaling up this mix and also for the subsequent analyses we assume an adaptation delay of five years during which the absorption capacity for the interventions is gradually implemented. The final share of malaria budget on total GDP is reached in the year 2017 and maintained either through 2025 or 2035, depending on the scenario.

Cost calculations only consider the direct costs such as the price per IVM intervention per person per year (Table S1) and the direct benefits such as impacts on gross domestic production (GDP). Indirect or external costs of DDT, i.e., costs related to environmental and health impacts are difficult to assess. This is due both to the lack of sufficient data on these impacts and also to the fact that the trade off between short term epidemiological improvements due to DDT and the long term external costs involves value judgments and depends on risk attitudes [7]. We add indications of external costs and risks to highlight how they can affect evaluations of the effectiveness of the continued use of DDT for IRS versus its rapid phase out. The use of DDT in IRS is for example often linked to fears that DDT can threaten agricultural exports (e.g., [36]) either because of actual contamination of food crops or because of consumer concerns mainly in European countries. Although DDT is applied indoors, dwellings in rural settings are often very close to farmed areas so that DDT spillovers to crops become possible and unavoidable if one considers the eventual decay of sprayed walls. The risks of actual contamination of food crops are very difficult to quantify. As an alternative to quantifying these risks we compare the difference in the costs of the continued use of DDT for IRS versus its rapid phase out to the value of the agricultural exports in SSA.

The simulation model also contains a climate-suitability index (see Figure S2) that allows analyzing different climate change scenarios. In this paper, however, we focus on the policy-scenario combinations summarized Table 1 and retain climate change analyses for further research.

## Results

This section describes results obtained from simulating our baseline scenario and comparing different policy and scenario analyses to this baseline. It is important to note that the simulation results presented for malaria and other key socio-economic variables cannot be interpreted as precise forecasts. Instead, they are indications of the likely development of the system under the current and under modified policy frameworks.

### Baseline and model validation

Within this section we discuss simulation results and historical data for the baseline scenario. The baseline simulation shows a close fit between historical data and model results for the major socio-economic indicators (R^2^ of almost 1 for population development, 0.995 for average adult literacy rate, 0.79 for average life expectancy and 0.969 for GDP). The simulated value of economic production (GDP) very well matches historical data, indicating that the extended Cobb-Douglas production function can reasonably explain observed economic growth. A further indication of model validity is that the simulated under-five mortality rate in the future is in line with the United Nations' population projections [37].

Baseline projections into the future depict an s-shaped increase in population and a continuous, albeit slow growth in literacy rate and life expectancy which is partly due to the steadily increasing population that makes it more difficult for education and health interventions to reach the entire target population. GDP is projected to increase continuously as a consequence of improvements in education and health. The growth in per capita GDP is slower as the total GDP has to be distributed among a steadily increasing population.

Key malaria indicators such as the total estimated malaria cases (Figure S3) depend on the ecological as well as socio-economic context and on the available funding for malaria control (government funding plus external funding). These funds were 0.27% of total GDP in 2008, the last year with available statistical data, and are assumed to grow by 1% each year for baseline simulations into the future. Historical development of malaria cases, i.e., of the number of malaria infected people per year, is not well documented. Official data from the WHO covering most of the SSA region is available only starting in the 1990s (World Malaria Reports from several years). Even for that period, data is characterized by several holes (missing data from reporting countries), and it only represents reported cases, which are estimated to be a small fraction of actual cases [38]. Estimated malaria cases seem to have steadily increased until the 2000s and have since experienced a considerable decline that can be explained by the large investments – especially concerning the distribution of bed nets – operated by the major malaria programs between 2000 and 2010. The model replicates this trend (Figure S3). The steady increase in malaria cases prior to 2000 is a consequence of the growing population in SSA and the fact that IVM coverage between 1970 and 2000 was approximately constant. The oscillations in the historical time period, both for total estimated malaria cases as well as the population fraction affected by malaria, is a consequence of identical oscillatory patterns in the climate suitability index.

For the next two decades, the model projects a fairly stable number of malaria cases (around 360 Million), followed by a significant decline which brings the number of cases close to 200 Million in 2050 (Figure S3). The overall decrease in malaria cases is due to the continuation of current malaria control policies, most importantly of prevention policies. A continuous increase in the coverage of IVM interventions first leads to a stabilization of the (otherwise rapidly growing) vulnerable population, and eventually to its decline. This is also reflected in the declining fraction of the population that is affected by malaria (Figure S3, solid black line).

### Economic costs of malaria

For calculating the economic costs of malaria, we ran a simulation that assumed no malaria as of 2012. In this counterfactual exercise, development would only be determined by the interaction between the four non-malaria model sectors population, education, health, and production. By comparing this simulation to the baseline values we can estimate the losses in GDP due to malaria (difference between the no malaria scenario and the baseline) According to this calculation, the losses in GDP due to malaria in 2012 are approximately 11 Billion US $. This value is almost identical to the costs of malaria calculated in the Global Malaria Action Plan [39]. At the end of the simulation period, the GDP loss is approximately 22 billion US $, assuming a discount rate of 3%.

### Costs and benefits of rapidly phasing out DDT

In this section we address the central research question of this paper, i.e., the costs and benefits of a continued use of DDT for IRS versus its rapid phase out.

#### Direct economic costs and benefits of rapidly phasing out DDT for IRS

The direct economic costs of non-DDT insecticides are approximately 50% higher than those of DDT (Table S1 and quoted sources). Figure S4 compares the total average yearly expenditures for the current mix policy (2035-CurrentMix) and the current mix without DDT policy (2035-NoDDT), both of which target malaria elimination by 2035. The figure also indicates the necessary additional malaria expenditures as percentage of GDP required to eliminate malaria by 2035 for the two scenarios (percentage values in brackets; 0.178% of GDP for 2035-CurrentMix and 0.199% for 2035-NoDDT). All expenditure figures assume a discount rate of 3%. The figure shows that malaria elimination by 2035 without the use of DDT entails higher total average yearly expenditures than the current mix scenario, which could be expected from the difference in insecticide costs.

The estimated difference in costs between DDT and other IRS is based on the conservative assumption that the difference in production costs will remain unchanged, while it is likely that a large increase in the production of non-DDT IRS products will lead to a reduction in their cost.

The average yearly costs for eliminating malaria by 2035 with the current combination of interventions and either a continued use of DDT for IRS or a rapid phase out of DDT for IRS can be compared with the yearly gains in GDP that result from malaria elimination.

The continuation of DDT for IRS and a rapid phase out of DDT for IRS do not differ significantly in terms of the yearly gain in GDP that can be realized by the two policies. Both policies gradually approach the full yearly gains in GDP over time. Both policies stabilize on a percentage difference of approximately 1.6%, i.e., they appropriate most of the GDP loss due to malaria and thus realize 98.4% of the full potential yearly GDP gains. The remaining difference to the full potential GDP gains is due to the fact that these policies only gradually approach malaria elimination. The hypothetical no malaria scenario, on the other hand, assumes no malaria as of 2012 and thus benefits from a more productive labor force as of 2012 as well as from the allocation of funds that would otherwise go to malaria prevention and treatment to economically more productive purposes such as investments in education and health.

#### Indications of risks associated with the continued use of DDT

A contamination of agricultural products with DDT might imply losses in agricultural exports. Based on the data used in the production sector of the MMM, agricultural exports outside sub Saharan Africa have historically been around 0.1 percent of GDP. For our simulation runs into the future we therefore also assume agricultural exports outside SSA to be 0.1% of GDP. Figure S5 shows the average yearly value of 1%, 5% and 10% of the total agricultural exports (light grey bars) for the time period between 2017 and 2035, assuming a discount rate of 3%. This time period is equal to the time period for which the figure shows the average additional yearly costs of the 2035-NoDDT policy (as compared to the 2035-CurrentMix policy; dark grey bar).

Figure S5 shows that the difference in costs of the continued use of DDT for IRS versus its rapid phase out lie between 1% and 5% of the value of agricultural exports outside SSA. This comparison can inform an assessment of the risk of losing agricultural exports because it provides an estimate of how the additional direct costs of rapidly phasing out DDT relate to the value of agricultural exports outside SSA. For each country in SSA this risk assessment is likely to be different as the importance of agricultural exports differs considerably.

#### Indications of external effects of the continued use of DDT

In addition to the purely economic direct costs of phasing out DDT there are also possible external costs of DDT for human health and the environment. These costs are difficult to quantify because of the ecosystem-wide diffusion of DDT and the long-term nature of its manifold impacts. The most evident threat to human health is the direct exposure of humans to DDT on dwellings' walls. Based on our simulations, total DDT on dwellings is expected to grow up to nearly 90'000 tons in 2050 in our 2035-CurrentMix policy (dashed grey line in Figure S6). This value results from scaling up the current amount of DDT used for IRS and includes DDT concentrations that were accumulated on dwellings prior to 2010. On the contrary, in our 2035-NoDDT policy (solid grey line), DDT levels on dwellings are projected to decrease and reach levels very close to zero by 2020.

### Analyses of different intervention mixes and target years

For putting the key question about the costs and benefits of a continued use of DDT for IRS versus its rapid phase out into perspective, we calculate the same costs and benefits for alternative combinations of IVM interventions. In this section we therefore calculate the malaria expenditures necessary to gradually scale up the current and alternative combinations of IVM interventions for achieving malaria elimination in 2025 or 2035. In addition to calculating the costs of elimination we compare these costs to the gain in GDP that could be achieved through malaria elimination.

In order to eliminate malaria by 2025 with the current mix of interventions, the additional share of malaria expenditure on GDP needs to be 0.219% between 2017 and 2025. The total average yearly costs (malaria prevention expenditure) of this policy are 1.18 billion US dollars. Figure S7 summarizes the average yearly costs for all our policy-scenario combinations and arranges them in order of increasing costs. The figure also indicates the additional share of malaria expenditure on GDP for each policy-scenario combination in the brackets at the bottom of the bars. Figure S7a shows the average yearly costs for eliminating malaria by 2025 and Figure S7b the average yearly costs for the 2035 scenario.

Figure S7 shows a clear order of intervention mixes in terms of their costs. Mixes focusing on the use of bed nets and on environmental management are cheaper than the currently applied mix, which, in terms of costs, is superior to a mix focusing on IRS. The mix focusing on IRS is the most expensive mix. This is mainly caused by the fact that IRS implies recurrent expenditure and does not have an important capital component. The bed net and EM mixes, on the other hand, require developing IVM infrastructure such as the production facilities for bed nets and, in case of EM, the construction of small-scale water or house screening infrastructure which, once developed, only have to be maintained and keep producing socio-economic benefits over their lifetime. When compared to Figure S4, Figure S7b shows that most of the tested combinations of IVM interventions have lower direct costs than the current combination, either with a continued use of DDT for IRS (2035-CurrentMix) or with a rapid phase out of DDT for IRS (2035-NoDDT in Figure S4).

The average yearly costs of the different intervention mixes are also reflected in the necessary additional share of malaria expenditure on total GDP. The intervention mixes with 2025 as malaria elimination target year generally entail higher average yearly costs than the mixes for 2035. The 2025 interventions have to be more intensive to reach the shorter term goal than the 2035 interventions. Interestingly for the IRS focus, since the interventions are recurrent, average yearly expenditures are higher for elimination in 2035 than for elimination in 2025. Therefore, the later the elimination target is set to be reached, the higher the costs as the population base will have increased considerably in the additional malaria years.

These costs can be compared to the yearly gains in GDP resulting from malaria elimination. If malaria is eliminated by 2025, all intervention mixes (all policies) gradually and similarly approach an appropriation of the potential yearly gains in GDP of approximately 98.65% by 2025. In the 2035 scenarios, about 98.15% of the total potential gains are appropriated. As the 2025 scenarios eliminate malaria faster, they benefit from a more productive labor force earlier on and also from the allocation of expenditure previously used for malaria to more productive uses such as investments in education and health. The simulations with 2035 as target year for malaria elimination provide the interesting finding of faster gains in GDP resulting from the 2035-IRSFocus policy. As IRS does not require the installation of a capital component, expenditures become effective without major installation delays. Faster malaria improvements enable the economic benefits (more productive labor force and other investments) to materialize earlier.

These results proved to be fairly stable and insensitive to changes in the cost-effectiveness assumptions underlying ITN and IRS. A *first sensitivity simulation* analyzed the impact of variations in the cost-effectiveness of ITN, IRS and EM in a range of ±20% of the assumptions used for the previous simulations and reported in Table S1. The simulation used the total malaria prevention expenditure in the 2025-CurrentMix policy. Sensitivity analysis showed that under the most cost-effective assumptions, malaria could be eliminated about four years earlier. Decreases in cost-effectiveness, however, delay malaria elimination up to nine years. This asymmetry around the year 2025 is caused by the adaptation delay of five years during which the absorption capacity for the interventions is gradually implemented and which prevents much faster elimination even under very high cost-effectiveness of IVM interventions.

A *second round of sensitivity simulations* decreased the cost-effectiveness of ITN by 20%, thus taking into account new evidence on reduced ITN effectiveness [40]. At the same time, it increased the cost-effectiveness of IRS by 20% to make ITN even less competitive. With these assumptions, we ran the 2025-policies again. Even under these modified assumptions, the policies maintained their order in terms of overall cost-effectiveness, i.e., the ITN focus mix followed by EM focus, current mix and eventually IRS mix. This must be due to the fact that all combinations of IVM interventions have a considerable ITN component (33 to 66%, see Table 1). The results presented in Figure S7 thus prove to be reasonably stable over a wide range of cost-effectiveness assumptions.

### Summary of results in light of the research questions

At the beginning of this paper we addressed one central research question and four additional questions necessary for putting the central question about the costs and benefits of a continued use of DDT for IRS versus its rapid phase out into perspective. With the results from the simulations with the Malaria Management Model (MMM) we can summarize the answers to these questions as below. Analysis of the baseline scenario (a projection under the current policy framework) indicated an overall improvement in malaria control – although not a very rapid one – with a reduction of malaria cases to about 200 million in 2050. Such results are not to be intended as point-predictions, but rather as the likely tendency if no major shift in policy regime were to take place.

#### Central question: What are the costs and benefits associated with the continued use of DDT for IRS in sub Saharan Africa versus its rapid phase out?

The direct economic costs of non-DDT insecticides are approximately 50% higher than those of DDT and the total direct costs for eliminating malaria by 2035 with the current combination of IVM interventions and a rapid phase out of DDT for IRS are about 12% higher than the total costs of the same combination of interventions with a continued use of DDT for IRS. A gradual reduction in the price of non-DDT IRS products would further reduce this cost difference. A rapid phase out of DDT, however, creates a series of benefits as well as avoided risks that have the potential of exceeding the additional costs. Phasing out of DDT would avoid the risk of losing some of the agricultural exports outside the SSA region. A loss of exports in the range between 1 and 5% is in the same range as the costs of substituting DDT. Other benefits include health and environmental effects that are difficult to measure.

#### Additional questions: What is the amount of resources necessary to gradually scale up the current or alternative combinations of vector control interventions for achieving malaria elimination in 2025 or in 2035, respectively? How does this amount compare to the gain in GDP that could be achieved through malaria elimination?

For all intervention mixes the necessary additional malaria expenditure as a share of total GDP for the time period between now and either 2025 or 2035 (depending on the scenario) needs to be in the range of 0.17 and 0.24 percent to enable elimination. The total average annual costs are around 1.2 Billion US $ (real terms, base 2000), assuming a 3% discount rate. In our policy-scenario tests we found a fairly stable rank order of intervention mixes in terms of their ratio between additional value added by malaria elimination and the costs for achieving this. The stability of this order to variations in cost-effectiveness assumptions is encouraging for an aggregated model such as MMM that requires simplification of the represented processes. All of the tested policy-scenario combinations showed that the average yearly expenditures for malaria prevention were much lower than the possible yearly gain in GDP from eliminating malaria (economic costs of malaria calculated in the hypothetical no malaria scenario). Scaling up funding for malaria control interventions thus seems to be more than viable even from a narrow, purely economic perspective.

In terms of the average yearly expenditures, a mix favoring the use of ITN clearly exceeded all other intervention mixes, assuming that the expected improvements in education levels in the future will empower beneficiaries to use bed nets more appropriately than today and thus support the leading role of ITN among IVM interventions. Intervention mixes focusing on environmental management also exhibited fairly low average yearly costs. This supports findings that highlight the importance of EM as a supplement to ITN [41], [42]. It is, however, unclear how broadly these interventions are applicable, i.e., how much they can be scaled up from the current situation [4], [43], [44],). Finally, IRS interventions are the most expensive interventions. A relevant issue regarding the effectiveness of IRS is the mounting resistance of mosquitoes to the sprayed chemicals [45]. Combinations of IVM interventions with a high share of IRS are more expensive than other combinations. However, IVM interventions with a high share of IRS create faster gains in GDP. These analyses reveal a trade off between cost minimization and benefit maximization.

## Discussion

The purpose of this paper was to test the Malaria Management Model (MMM) in a pilot application that provides a differentiated assessment of the costs and benefits of the continued use of DDT for IRS versus its rapid phase. The Malaria Management Model is a prototype of a computer based scenario analysis tool that integrates malaria transmission, case management as well as Integrated Vector Management into a socio-economic development framework for the case of the aggregated sub Saharan African region. The model studies long-term (1970–2050) trends of malaria diffusion in sub Saharan Africa, the implications for socio-economic development, and it compares the cost and effectiveness of alternative malaria control strategies in the long run.

Our simulations showed that the direct economic costs of a continued use of DDT for IRS are lower than for a rapid phase out of DDT for IRS. However, we were also able to quantify indications about external costs of DDT and economic risks that can be avoided with a rapid phase out of DDT for IRS. Simulation runs with alternative combinations of IVM interventions also demonstrated that comparatively less costly combinations exist to the current combination of IVM interventions or a combination with an even stronger focus on IRS.

The use of malaria models has been advocated repeatedly as tools for strategic planning, development of management plans, impact assessment, technical feasibility assessments, and operational feasibility assessments [46]. Our simulation model complements models serving the first purpose, strategic planning. It provides an innovative, integrated approach to study malaria diffusion and control strategies for the aggregated SSA region in the long run. Our simulations complement existing findings with the feature of testing several policy-scenario combinations and thus putting the central question of the costs and benefits of phasing out DDT for IRS into perspective. Such tests allow exploring the range of possible outcomes, in our case particularly the costs for eliminating malaria, and the stability of the costs for different intervention mixes under different scenarios (target years for eliminating malaria). The endogenous representation of variables such as population and GDP allows calculating aspects of costs and benefits of the policy-scenario combinations over time. This is particularly important as the target (malaria elimination) becomes more difficult to achieve, the later interventions are implemented and thus the more the population that needs to be covered by the interventions has grown (see also [21].

The MMM with its capacity to compare some of the costs and benefits of different policy-scenario combinations is helpful to policymakers, e.g., in providing orientation and stimulating discussions when the continued need for DDT in IRS is re-evaluated by the Stockholm Convention every two years. Comparing the costs for eliminating malaria and the economic benefits of doing so (i.e., the gain in GDP) for different combinations of vector control interventions provides important decision support for actors in global health activities. The MMM can also contribute to raising and maintaining awareness with development partners that all strategies aiming at eliminating and eventually eradicating malaria need to be sustained over time periods that exceed the usual organizational or political planning horizons. Awareness raising also concerns the danger involved in premature reductions in expenditures for malaria elimination and eradication which would severely compromise improvements in the malaria situation achieved until this point in time.

In addition to providing quantitative results, the MMM is also an analytical framework that allows careful analyses of policy alternatives proposed by actors in the field or resulting from further research. Simulations and sensitivity analyses allow decision makers to explore effects of such policies on a range of outcomes over time. They also identify competing objectives such as, in the context of this paper, the minimization of direct costs of IVM interventions versus the minimization of adverse long term effects of the use of DDT. Simulation models help decision makers to confront such competing objectives or trade offs by separating issues of scientific uncertainty (e.g., the impact of DDT on human health and the environment) from disagreement over competing objectives. Issues of scientific uncertainty can be subjected to sensitivity analysis so that the impact of different assumptions can be visualized. The MMM as an example of a simulation model thus provides a user-friendly tool that creates more systematic mechanisms for analyzing alternative interventions and making informed trade offs.

### Limitations of the approach

The integrated nature of our approach necessarily entails simplifications and uncertainties in many ways. Malaria epidemiology is highly aggregated and the model cannot evaluate the most effective mix for eliminating malaria. The most severe data limitations are the data on malaria cases which have a high degree of uncertainty. This makes it difficult to calibrate the entire model and particularly the malaria transmission subsector to the data. There is also some uncertainty about the unit costs of the IVM interventions, particularly for EM and IRS. Unit costs can be subject to economies of scale, diminishing returns or increasing costs e.g., for reaching more remote population in rural areas. In the case of IRS, the current evidence is even insufficient to quantify properly the effect of IRS in high transmission settings [47]. The model also does not consider the effect of combinations of ITN. However, as the difference between the costs for implementing scaled up IVM interventions and the gains in GDP is very big, uncertainty about the unit costs of IVM interventions does not affect the conclusions that can be drawn from the simulation results.

All our policy-scenario combinations are based on the assumption that current IVM interventions are continuously improved and further developed so that IVM can in fact be scaled up to the degree necessary for eliminating malaria. We also assume that (cost-effective) alternatives to DDT are not only feasible but that they can be scaled up to the level necessary for malaria elimination. While we explicitly represent constraints in the absorptive capacity of scaled up IVM interventions, our model does not address possible inefficiencies in the implementation of IVM interventions and it only describes a very aggregated process of building capacity for the effective implementation of IVM interventions and case management measures. The model can therefore not answer the question whether malaria elimination is really possible. It can only calculate the costs required for elimination in case the described assumptions hold.

Notwithstanding these uncertainties, simplifications and limitations of our approach, the costs estimated by our simulation model are in line with the costs estimated by the World Health Organization (WHO) [4] and our simulation model also calculated losses in GDP due to malaria that are almost identical to the estimates of Roll Back Malaria [2]. This is a strong indication of the validity of our results. The close fit between simulated data and historical data as recorded in statistical data sources further supports the validity of the simulation results.

### Further developments of the approach

Complementing the costs and benefits calculated by the simulation model requires further research. This concerns improvements of our database for malaria-related indicators and strengthening of our estimations of the effects of DDT on health and the environment. Such data would allow for more complete cost-benefit analyses and thus for more detailed decision support. Future applications of the MMM should also focus on climate change analyses which is particularly relevant because changing rain patterns will considerably affect malaria occurrence until 2050. They will also affect migration of people and land use and as such further alter the occurrence of malaria. Such analyses could test the robustness of the calculations presented in this paper for different climate change scenarios. The model in its current form already incorporates features (climate suitability index) that allow for such analyses.

In this paper we have described the pilot application of the MMM approach to the aggregated SSA region. Further applications of the MMM model should focus on country-specific analyses. This requires more detailed data about the malaria context, environmental conditions and social factors. Given the availability of data, the MMM can easily be applied to the national level where it is possible to provide much more detailed and specific decision support in the assessment and evaluation of different malaria control interventions.

## Supporting Information

Figure S1 **Aggregated representation of the MMM model.** Aggregated representation of the structure of the Malaria Management Model. The structure is composed of five sectors: population, production, education, health, and malaria. All sectors interact with each other and the arrows between the sectors describe the directions of these interactions. The malaria sector is itself split into five subsectors.
(TIF)

Figure S2 **Malaria subsectors describing transmission, IVM and case management.** Overview of the malaria subsectors in the malaria management model. Variables in *italics* are variables that enter the malaria subsectors from the four socio-economic sectors. The transmission subsector describes the process during which the vulnerable population can actually be infected with and die from malaria. Infections depend on the coverage with IVM interventions and the model assumes that no infections occur when the entire vulnerable population is effectively covered by IVM interventions (subsector IVM interventions). Malaria deaths can be prevented by covering the infected population with effective treatment measures (subsector case management).
(TIF)

Figure S3 **Baseline simulations for total estimated malaria cases (solid grey line) and population fraction affected by malaria (solid black line).** Baseline simulations for malaria cases. Total estimated malaria cases (grey line, million people) increased steadily until the 2000s (with oscillations that follow oscillations in the climate suitability index) and have since experienced a considerable decline that can be attributed to large investments made as a consequence renewed interest in malaria eradication. Total malaria cases are projected to stabilize and decline as a consequence of increases in IVM coverage that come with increases in GDP as well as improvements in education and health. The population fraction affected by malaria (black line; i.e., the proportion of the total population affected by malaria) is projected to decline even more as total malaria cases stabilize while the total population experiences further growth in the baseline projections for the future.
(TIF)

Figure S4 **Average yearly costs for a continued use of DDT for IRS versus a rapid phase out of DDT for IRS.** Average yearly costs for a continued use of DDT for IRS versus its rapid phase out. When only direct costs such as the price per IVM intervention per year are considered the average necessary yearly expenditure for eliminating malaria by 2035 is lower for the current combination of IVM interventions using DDT for IRS (2035-CurrentMix) than for the same combination but with a rapid phase out of DDT for IRS (2035-NoDDT). Values in brackets describe the additional malaria expenditures as percentage of GDP required to eliminate malaria by 2035.
(TIF)

Figure S5 **Costs and benefits of rapidly phasing out DDT for IRS.** Costs and benefits of rapidly phasing out DDT for IRS. The direct costs of a rapid phase out of DDT are higher than the direct costs for the continued use of DDT for IRS (additional costs of the 2035-NoDDT policy; dark grey bar). The value of this difference is equivalent to something between 1% and 5% of the total agricultural exports outside sub Saharan Africa (light grey bars). This comparison can inform an assessment of the risk of losing agricultural exports because it provides an estimate of how the additional direct costs of rapidly phasing out DDT relate to the value of agricultural exports outside SSA.
(TIF)

Figure S6 **Comparison of model results for DDT on dwellings for baseline (black solid line), 2035-current mix (dashed grey line) and 2035-NoDDT simulations.** DDT concentrations on dwellings in three different scenarios. The most direct health threat of DDT in IRS results from the concentration of DDT on dwellings' walls. In the case of a continued use of DDT for IRS (2035-CurrentMix simulation), DDT concentrations increase steadily and considerably above the baseline values, where no malaria elimination is reached by 2035. In the case of a rapid phase out of DDT for IRS (2035-NoDDT simulation), DDT concentrations on dwellings decrease and approach levels close to zero after an extended adaptation delay.
(TIF)

Figure S7 **Average yearly costs for eliminating malaria with the different combinations of IVM interventions and for the two target years.** Average yearly costs for eliminating malaria with the different combinations of IVM interventions. The average yearly costs for eliminating malaria are higher in the case of 2025 as target elimination year. For both target elimination years, IVM interventions with a strong focus on ITN are the least costly combination of IVM interventions and interventions with a strong focus on IRS are the most expensive. This can be explained by the size of the capital component in the different combinations. Higher capital components require higher initial investments but then only need to be maintained. Recurrent expenditure as in the case of IRS, on the other hand, is equally high every year. Values in brackets describe the additional malaria expenditures as percentage of GDP required to eliminate malaria by 2025 or 2035, respectively.
(TIF)

Table S1 **Parameter values, assumptions and data sources for IVM interventions.** Notes: The number of people effectively covered by IVM interventions can be calculated as follows: - In the case of ITN: malaria prevention expenditure for ITN divided by the unit costs (the cost of one net) and multiplied by coverage (the number of people covered by one net). This term is adjusted for the effectiveness of the bed nets which depends in a linear way on the average years of schooling. 100% effectiveness would require that the average adult person has completed nine years of schooling. The effectiveness in 2010 is estimated to be 58%. - In the case of IRS: malaria prevention expenditure for IRS divided by unit costs and multiplied by effectiveness. As the current evidence is insufficient to quantify properly the effect of IRS in high transmission settings [47], we subject the cost-effectiveness assumptions to sensitivity analysis. - In the case of EM: malaria prevention expenditure for EM divided by the unit costs (costs per square kilometer) and multiplied by coverage (which depends in a nonlinear way on population density). - Total: The sum of the number of people covered by ITN, IRS and EM, adjusted for an overlapping factor of 50% (i.e., multiplied by a factor of 0.5). See references [49]–[66].
(DOC)

Text S1 **Technical appendix with all model equations.** The technical appendix lists all the equations used in the Malaria Management Model. Initial values and parameter values are those from the baseline simulation. The simulation model is also available as online supporting information (Dataset S1 and Dataset S2).
(DOC)

Dataset S1 **MMM model running in the Vensim ® software package.** This supporting information dataset is the Malaria Management Model that runs in the Vensim ® DSS software package. The model has to be completed with the data file (Dataset S2).
(MDL)

Dataset S2 **Dataset for MMM model to be added to the Vensim ® software package.** This supporting information dataset contains the data for the historical period of the simulation, i.e., for the years 1970 to 2010.
(VDF)
