# Supplementary material for: Application of the Malaria Management Model to the Analysis of Costs and Benefits of DDT versus Non-DDT Malaria Control
Source: PLoS One. 2011 Nov 30;6(11):e27771. doi: 10.1371/journal.pone.0027771 (PMC3227603; doi:10.1371/journal.pone.0027771)
Supplement: Table S1 — Parameter values, assumptions and data sources for IVM interventions. Notes: The number of people effectively covered by IVM interventions can be calculated as follows: - In the case of ITN: malaria prevention expenditure for ITN divided by the unit costs (the cost of one net) and multiplied by coverage (the number of people covered by one net). This term is adjusted for the effectiveness of the bed nets which depends in a linear way on the average years of schooling. 100% effectiveness would require that the average adult person has completed nine years of schooling. The effectiveness in 2010 is estimated to be 58%. - In the case of IRS: malaria prevention expenditure for IRS divided by unit costs and multiplied by effectiveness. As the current evidence is insufficient to quantify properly the effect of IRS in high transmission settings [47], we subject the cost-effectiveness assumptions to sensitivity analysis. - In the case of EM: malaria prevention expenditure for EM divided by the unit costs (costs per square kilometer) and multiplied by coverage (which depends in a nonlinear way on population density). - Total: The sum of the number of people covered by ITN, IRS and EM, adjusted for an overlapping factor of 50% (i.e., multiplied by a factor of 0.5). See references [49]–[66]. (DOC) [file pone.0027771.s008.doc]

| **IVM category** | **Variable** | **value** | **Unit** | **data sources** |
| --- | --- | --- | --- | --- |
| ITN | unit cost | 6 | $ | Estimation based on data covering a variety of SSA countries and international organizations working in the field, such as UNICEF, RBM, icipe, KEMRI, STI and others ([49], [50], [51]); in line with [39]. |
|  | coverage | 2 | person/net | [52] |
|  | average life (durability) | 5 | Year | [52] |
|  | effectiveness |  | % | depends linearly on the average years of schooling:  effectiveness 0 years: 25% ([53], [54], [55]); effectiveness 3 years: 50% ([56]); effectiveness 9 years: 100% (estimation based on [53], [54], [55] and evidence from [57]) |
| IRS-NON-DDT | unit cost | 6 | $/person/year | Based on [58] and [59]. We take average pyrethroid values as representative for non DDT-IRS as pyrethroids are by far the largest category of insecticides used ([11]). The unit costs include all program costs categories, not only the costs of the insecticide ([32]) |
|  | implementation delay | 1 | Year | Assuming that deployment requires more organization than for bed nets, and must be done at specific times in the year |
|  | effectiveness |  | % | Effectiveness 70% in non-resistance and unstable malaria areas based on [57] and [47]; adjusted for minimum resistance of 0%, which increases linearly to 100% for full coverage ([10]). |
| IRS-DDT | unit cost | 4 | $/person/year | Based on [58] and [60]. The unit costs include the costs of spray operations, of spray operations commodities, local labor and local administration, not only the costs of the insecticide ([32]). |
|  | implementation delay | 1 | Year | Assuming that deployment requires more organization than for bed nets, and must be done at specific times during the year. |
|  | effectiveness |  | % | Effectiveness 70% in non-resistance and unstable malaria areas based on [57] and [47]; adjusted for minimum resistance of 40%, which increases linearly to 100% for full coverage ([10]). |
| EM | EM unit construction cost | 3000 | $/km^2^ | Based on [61] and [62], assuming a 50% mix of small-scale water infrastructure and house screening |
|  | EM unit maintenance cost | 500 | $/km^2^/ year | [35] |
|  | EM coverage |  | % | Varying depending on population density (based on estimated population distribution table for the entire SSA region, obtained from National censuses and UN Pop) |
|  | EM average life | 8 | Year | [62], provided that infrastructure is properly maintained |
|  | implementation delay | 1 | Year | [34] |
|  | EM effectiveness | 35 | % | Based on [34], [31] for small-scale water infrastructure and [63], [64], [6] for house screening |
|  | larviciding unit cost | 1500 | $/km^2^/year | [7], [65] |
|  | larviciding coverage |  | % | Varying depending on population density (based on estimated population distribution table for the entire SSA region, obtained from National censuses and UN Pop) |
|  | larviciding effectiveness | 35 | % | [42], [41], [66] |
|  | mix EM – larviciding |  | % | 90% EM, 10% larviciding, gradual shift t0 50% by 2050. |
