# Supplementary material for: Application of the Malaria Management Model to the Analysis of Costs and Benefits of DDT versus Non-DDT Malaria Control
Source: PLoS One. 2011 Nov 30;6(11):e27771. doi: 10.1371/journal.pone.0027771 (PMC3227603; doi:10.1371/journal.pone.0027771)
Supplement: Text S1 — Technical appendix with all model equations. The technical appendix lists all the equations used in the Malaria Management Model. Initial values and parameter values are those from the baseline simulation. The simulation model is also available as online supporting information (Dataset S1 and Dataset S2). (DOC) [file pone.0027771.s009.doc]

## Technical appendix Text S9: complete list of model equations

Below we list all the equations used in the Malaria Management Model. Initial values and parameter values are those from the baseline simulation.

********************************

arrays

********************************

age: (AGE 0-AGE 79),AGE 80 AND OVER

region: SOUTH EAST, NORTH EAST, WEST, CENTRAL EAST, CENTRAL, OTHER

sex: FEMALE, MALE

********************************

Simulation control parameters

********************************

FINAL TIME = 2050

Units: Year

INITIAL TIME = 1970

Units: Year

TIME STEP = 0.0625

Units: Year

SAVEPER = 1

Units: Year

********************************

1 Population

********************************

AGE COHORT DURATION= 1

Units: Year

age group weight[sex, age group]= MAX(0,population group[sex,age group]/SUM(population group[sex,age group!]))

Units: Dmnl

age specific fertility distribution[childbearing age]= (indicated age specific fertility distribution [childbearing age]/cumulative fertility distribution)*REFERENCE CUMULATIVE FERTILITY DISTRIBUTION

Units: Dmnl/Year

AGE SPECIFIC FERTILITY DISTRIBUTION TABLE([(12,0)-(50,0.2)],(12,0),(17,0.0345071),(22,0.0546579),(27,0.046436),(32,0.033138),(37,0.0201312),(42,0.0085919),(47,0.002538))

Units: Dmnl/Year

aging[sex,age]= Population[sex,age]/AGE COHORT DURATION

Units: Person/Year

births[sex]= total fertility rate * SUM(sexually active women[childbearing age!]*age specific fertility distribution[childbearing age!])*proportion of babies by sex[sex]

Units: Person/Year

childbearing age woman= SUM(sexually active women[childbearing age!])

Units: Person

contraceptive prevalence= INITIAL CONTRACEPTIVE PREVALENCE*Effect Of Education On Contraceptive Prevalence

Units: Dmnl

cumulative fertility distribution= SUM(indicated age specific fertility distribution [childbearing age!])

Units: Dmnl/Year

DEATH RATES TABLE[FEMALE, AG UNDER 1]([(0,0)-(80,0.4)],(0,1),(20,0.31),(22.5,0.28),(25,0.26),(27.5,0.25),(30,0.23),(32.5,0.21),(35,0.2),(37.5,0.19),(40,0.17),(42.5,0.16),(45,0.15),(47.5,0.14),(50,0.13),(52.5,0.12),(55,0.11),(57.5,0.1),(60,0.09),(62.5,0.09),(65,0.08),(67.5,0.07),(70,0.06),(72.5,0.05),(75,0.04),(77.5,0.03),(80,0.02))DEATH RATES TABLE[FEMALE, AG 1 TO 4]([(0,0)-(80,0.2)],(0,1),(20,0.107747),(22.5,0.096214),(25,0.086166),(27.5,0.077297),(30,0.069386),(32.5,0.062265),(35,0.055811),(37.5,0.04992),(40,0.044518),(42.5,0.03942),(45,0.034525),(47.5,0.030069),(50,0.026002),(52.5,0.022273),(55,0.018843),(57.5,0.015675),(60,0.012736),(62.5,0.009978),(65,0.007747),(67.5,0.005892),(70,0.004318),(72.5,0.003019),(75,0.001988),(77.5,0.00121),(80,0.000663))

DEATH RATES TABLE[FEMALE, AG 5 TO 9]([(0,0)-(80,0.02)],(0,1),(20,0.018731),(22.5,0.016906),(25,0.015271),(27.5,0.013789),(30,0.01244),(32.5,0.011202),(35,0.01006),(37.5,0.009003),(40,0.00802),(42.5,0.006989),(45,0.006082),(47.5,0.005257),(50,0.004502),(52.5,0.003809),(55,0.003168),(57.5,0.002575),(60,0.00202),(62.5,0.001515),(65,0.001183),(67.5,0.000876),(70,0.000621),(72.5,0.000418),(75,0.000264),(77.5,0.000152),(80,7.8e-005))

DEATH RATES TABLE[FEMALE, AG 10 TO 14]([(0,0)-(80,0.01)],(0,1),(20,0.009836),(22.5,0.00892),(25,0.008096),(27.5,0.007347),(30,0.006662),(32.5,0.006034),(35,0.005453),(37.5,0.004914),(40,0.004411),(42.5,0.003941),(45,0.003472),(47.5,0.00304),(50,0.002642),(52.5,0.002272),(55,0.001927),(57.5,0.001609),(60,0.001309),(62.5,0.001026),(65,0.000809),(67.5,0.000617),(70,0.000452),(72.5,0.000316),(75,0.000208),(77.5,0.000128),(80,7e-005))

DEATH RATES TABLE[FEMALE, AG 15 TO 19]([(0,0)-(80,0.02)],(0,1),(20,0.013531),(22.5,0.012284),(25,0.011165),(27.5,0.01015),(30,0.009223),(32.5,0.008369),(35,0.007584),(37.5,0.006853),(40,0.006174),(42.5,0.005598),(45,0.004943),(47.5,0.004339),(50,0.003783),(52.5,0.003269),(55,0.002794),(57.5,0.002351),(60,0.001937),(62.5,0.001549),(65,0.001173),(67.5,0.000896),(70,0.000659),(72.5,0.000462),(75,0.000306),(77.5,0.000186),(80,0.000102))

DEATH RATES TABLE[FEMALE, AG 20 TO 24]([(0,0)-(80,0.02)],(0,1),(20,0.01693),(22.5,0.015376),(25,0.013979),(27.5,0.012715),(30,0.011562),(32.5,0.010504),(35,0.009528),(37.5,0.008621),(40,0.00778),(42.5,0.00704),(45,0.00625),(47.5,0.005517),(50,0.004839),(52.5,0.004207),(55,0.00362),(57.5,0.003071),(60,0.002559),(62.5,0.002075),(65,0.001607),(67.5,0.001247),(70,0.000934),(72.5,0.000669),(75,0.000452),(77.5,0.000286),(80,0.000162))

DEATH RATES TABLE[FEMALE, AG 25 TO 29]([(0,0)-(80,0.02)],(0,1),(20,0.01819),(22.5,0.016536),(25,0.015052),(27.5,0.013709),(30,0.012484),(32.5,0.011361),(35,0.010323),(37.5,0.009362),(40,0.008468),(42.5,0.0077),(45,0.006864),(47.5,0.006088),(50,0.005365),(52.5,0.004692),(55,0.004063),(57.5,0.003476),(60,0.002925),(62.5,0.002404),(65,0.001875),(67.5,0.00147),(70,0.001114),(72.5,0.000811),(75,0.000559),(77.5,0.000358),(80,0.00021))

DEATH RATES TABLE[FEMALE, AG 30 TO 34]([(0,0)-(80,0.02)],(0,1),(20,0.018977),(22.5,0.017253),(25,0.015708),(27.5,0.01431),(30,0.013033),(32.5,0.011864),(35,0.010784),(37.5,0.009786),(40,0.008855),(42.5,0.008013),(45,0.007182),(47.5,0.006403),(50,0.005674),(52.5,0.00499),(55,0.004346),(57.5,0.003738),(60,0.003168),(62.5,0.002626),(65,0.002147),(67.5,0.001714),(70,0.001326),(72.5,0.000986),(75,0.000697),(77.5,0.000462),(80,0.00028))

DEATH RATES TABLE[FEMALE, AG 35 TO 39]([(0,0)-(80,0.02)],(0,1),(20,0.019955),(22.5,0.018173),(25,0.016574),(27.5,0.015127),(30,0.013806),(32.5,0.012597),(35,0.011481),(37.5,0.010448),(40,0.009486),(42.5,0.008628),(45,0.00778),(47.5,0.006981),(50,0.006227),(52.5,0.005519),(55,0.004847),(57.5,0.004215),(60,0.003616),(62.5,0.003047),(65,0.002527),(67.5,0.002046),(70,0.001611),(72.5,0.001223),(75,0.000886),(77.5,0.000603),(80,0.000378))

DEATH RATES TABLE[FEMALE, AG 40 TO 44]([(0,0)-(80,0.04)],(0,1),(20,0.020193),(22.5,0.018461),(25,0.01691),(27.5,0.015505),(30,0.014225),(32.5,0.013048),(35,0.011965),(37.5,0.01096),(40,0.010027),(42.5,0.009187),(45,0.008396),(47.5,0.00764),(50,0.006917),(52.5,0.006229),(55,0.005574),(57.5,0.004949),(60,0.004356),(62.5,0.003785),(65,0.003247),(67.5,0.002717),(70,0.002218),(72.5,0.001756),(75,0.001338),(77.5,0.000966),(80,0.000653))

DEATH RATES TABLE[FEMALE, AG 45 TO 49]([(0,0)-(80,0.04)],(0,1),(20,0.021268),(22.5,0.019524),(25,0.01796),(27.5,0.016544),(30,0.015252),(32.5,0.014068),(35,0.012976),(37.5,0.011965),(40,0.011022),(42.5,0.010177),(45,0.009401),(47.5,0.008657),(50,0.007937),(52.5,0.007244),(55,0.00658),(57.5,0.005944),(60,0.005333),(62.5,0.004745),(65,0.004171),(67.5,0.003582),(70,0.00301),(72.5,0.002464),(75,0.001952),(77.5,0.001478),(80,0.001056))

DEATH RATES TABLE[FEMALE, AG 50 TO 54]([(0,0)-(80,0.04)],(0,1),(20,0.027763),(22.5,0.025529),(25,0.023525),(27.5,0.021717),(30,0.020072),(32.5,0.018562),(35,0.017174),(37.5,0.015889),(40,0.014693),(42.5,0.013557),(45,0.012614),(47.5,0.011701),(50,0.010815),(52.5,0.009956),(55,0.009127),(57.5,0.00833),(60,0.007561),(62.5,0.006851),(65,0.006135),(67.5,0.005361),(70,0.004602),(72.5,0.003858),(75,0.00314),(77.5,0.002458),(80,0.001829))

DEATH RATES TABLE[FEMALE, AG 55 TO 59]([(0,0)-(80,0.04)],(0,1),(20,0.038124),(22.5,0.03506),(25,0.032325),(27.5,0.029863),(30,0.027627),(32.5,0.025582),(35,0.023703),(37.5,0.021968),(40,0.02036),(42.5,0.018867),(45,0.017601),(47.5,0.016369),(50,0.015178),(52.5,0.014022),(55,0.012907),(57.5,0.011831),(60,0.010795),(62.5,0.009813),(65,0.008829),(67.5,0.007759),(70,0.006703),(72.5,0.005664),(75,0.004653),(77.5,0.003683),(80,0.002777))

DEATH RATES TABLE[FEMALE, AG 60 TO 64]([(0,0)-(80,0.08)],(0,1),(20,0.063586),(22.5,0.058245),(25,0.05352),(27.5,0.049301),(30,0.045498),(32.5,0.042044),(35,0.038887),(37.5,0.035987),(40,0.033308),(42.5,0.030763),(45,0.028708),(47.5,0.026711),(50,0.024778),(52.5,0.022906),(55,0.021102),(57.5,0.019361),(60,0.017689),(62.5,0.016171),(65,0.014587),(67.5,0.012867),(70,0.01116),(72.5,0.009478),(75,0.007832),(77.5,0.006246),(80,0.004753))

DEATH RATES TABLE[FEMALE, AG 65 TO 69]([(0,0)-(80,0.1)],(0,1),(20,0.098693),(22.5,0.090243),(25,0.082871),(27.5,0.076358),(30,0.070546),(32.5,0.065312),(35,0.060565),(37.5,0.056235),(40,0.05226),(42.5,0.048607),(45,0.045604),(47.5,0.042686),(50,0.039859),(52.5,0.037122),(55,0.034479),(57.5,0.031933),(60,0.029484),(62.5,0.027191),(65,0.024787),(67.5,0.022148),(70,0.019498),(72.5,0.016848),(75,0.014214),(77.5,0.011621),(80,0.009119))

DEATH RATES TABLE[FEMALE, AG 70 TO 74]([(0,0)-(80,0.2)],(0,1),(20,0.159395),(22.5,0.145124),(25,0.133009),(27.5,0.12254),(30,0.113358),(32.5,0.105221),(35,0.097937),(37.5,0.091361),(40,0.08539),(42.5,0.080084),(45,0.075702),(47.5,0.071445),(50,0.067315),(52.5,0.063316),(55,0.059453),(57.5,0.055731),(60,0.052151),(62.5,0.048695),(65,0.045019),(67.5,0.040933),(70,0.036768),(72.5,0.032528),(75,0.028216),(77.5,0.023862),(80,0.019513))

DEATH RATES TABLE[FEMALE, AG 75 TO 79]([(0,0)-(80,0.4)],(0,1),(20,0.252331),(22.5,0.227439),(25,0.207422),(27.5,0.190805),(30,0.176687),(32.5,0.164479),(35,0.153767),(37.5,0.144268),(40,0.135763),(42.5,0.128251),(45,0.122317),(47.5,0.116549),(50,0.110954),(52.5,0.105536),(55,0.100302),(57.5,0.095255),(60,0.090401),(62.5,0.085765),(65,0.08059),(67.5,0.074756),(70,0.068712),(72.5,0.062434),(75,0.055897),(77.5,0.049101),(80,0.042068))

DEATH RATES TABLE[FEMALE, AG 80 AND OVER]([(0,0)-(80,0.6)],(0,1),(20,0.410881),(22.5,0.389394),(25,0.370157),(27.5,0.352756),(30,0.336902),(32.5,0.322367),(35,0.308965),(37.5,0.296544),(40,0.284989),(42.5,0.274432),(45,0.265855),(47.5,0.257306),(50,0.248818),(52.5,0.240404),(55,0.232093),(57.5,0.223906),(60,0.215861),(62.5,0.208031),(65,0.199094),(67.5,0.188776),(70,0.1778),(72.5,0.166073),(75,0.15349),(77.5,0.139955),(80,0.125409))

DEATH RATES TABLE[MALE, AG UNDER 1]([(0,0)-(80,0.4)],(0,1),(19.92,0.34),(22.299,0.311),(24.661,0.289),(27.007,0.269),(29.321,0.251),(31.636,0.234),(33.95,0.218),(36.233,0.204),(38.501,0.19),(40.634,0.178),(42.861,0.167),(45.118,0.156),(47.372,0.145),(49.623,0.135),(51.869,0.125),(54.108,0.115),(56.341,0.106),(58.58,0.097),(61.252,0.086),(63.659,0.076),(66.08,0.066),(68.524,0.056),(70.993,0.047),(73.486,0.037),(76.002,0.028))

DEATH RATES TABLE[MALE, AG 1 TO 4]([(0,0)-(80,0.2)],(0,1),(19.92,0.101096),(22.299,0.090657),(24.661,0.08153),(27.007,0.073448),(29.321,0.066219),(31.636,0.059702),(33.95,0.053783),(36.233,0.048373),(38.501,0.043402),(40.634,0.039138),(42.861,0.034408),(45.118,0.030108),(47.372,0.026188),(49.623,0.0226),(51.869,0.019304),(54.108,0.016268),(56.341,0.013454),(58.58,0.010822),(61.252,0.007944),(63.659,0.00606),(66.08,0.00446),(68.524,0.003132),(70.993,0.002074),(73.486,0.00127),(76.002,0.000701))

DEATH RATES TABLE[MALE, AG 5 TO 9]([(0,0)-(80,0.02)],(0,1),(19.92,0.01657),(22.3,0.015023),(24.66,0.013635),(27.01,0.012379),(29.32,0.011231),(31.64,0.010177),(33.95,0.009206),(36.23,0.008305),(38.5,0.007467),(40.63,0.006619),(42.86,0.005848),(45.12,0.005134),(47.37,0.004476),(49.62,0.003864),(51.87,0.003296),(54.11,0.002767),(56.34,0.00227),(58.58,0.001802),(61.25,0.001382),(63.66,0.001058),(66.08,0.000783),(68.52,0.000553),(70.99,0.000366),(73.49,0.000226),(76,0.000126))

DEATH RATES TABLE[MALE, AG 10 TO 14]([(0,0)-(80,0.008)],(0,1),(19.92,0.007768),(22.3,0.007094),(24.66,0.006486),(27.01,0.005934),(29.32,0.005429),(31.64,0.004963),(33.95,0.004533),(36.23,0.004134),(38.5,0.003762),(40.63,0.003411),(42.86,0.003081),(45.12,0.002771),(47.37,0.002478),(49.62,0.0022),(51.87,0.001937),(54.11,0.00169),(56.34,0.001454),(58.58,0.001231),(61.25,0.001008),(63.66,0.000819),(66.08,0.000645),(68.52,0.00049),(70.99,0.000356),(73.49,0.000244),(76,0.000154))

DEATH RATES TABLE[MALE, AG 15 TO 19]([(0,0)-(80,0.02)],(0,1),(19.92,0.011745),(22.3,0.010717),(24.66,0.009792),(27.01,0.008955),(29.32,0.008187),(31.64,0.007483),(33.95,0.006833),(36.23,0.006229),(38.5,0.005666),(40.63,0.005183),(42.86,0.004671),(45.12,0.004191),(47.37,0.003738),(49.62,0.003312),(51.87,0.002911),(54.11,0.002533),(56.34,0.002175),(58.58,0.001835),(61.25,0.001484),(63.66,0.001197),(66.08,0.000936),(68.52,0.000705),(70.99,0.000507),(73.49,0.000342),(76,0.000212))

DEATH RATES TABLE[MALE, AG 20 TO 24]([(0,0)-(80,0.02)],(0,1),(19.92,0.017833),(22.3,0.016267),(24.66,0.014861),(27.01,0.013588),(29.32,0.012429),(31.64,0.011363),(33.95,0.010379),(36.23,0.00947),(38.5,0.008621),(40.63,0.007904),(42.86,0.007154),(45.12,0.006397),(47.37,0.00569),(49.62,0.00503),(51.87,0.004413),(54.11,0.003833),(56.34,0.00329),(58.58,0.002775),(61.25,0.002155),(63.66,0.001702),(66.08,0.001313),(68.52,0.000974),(70.99,0.000687),(73.49,0.000454),(76,0.000274))

DEATH RATES TABLE[MALE, AG 25 TO 29]([(0,0)-(80,0.02)],(0,1),(19.92,0.017861),(22.3,0.016286),(24.66,0.014872),(27.01,0.01359),(29.32,0.012423),(31.64,0.011351),(33.95,0.01036),(36.23,0.009445),(38.5,0.00859),(40.63,0.007869),(42.86,0.007098),(45.12,0.006369),(47.37,0.005682),(49.62,0.005036),(51.87,0.004429),(54.11,0.003856),(56.34,0.003316),(58.58,0.002804),(61.25,0.00226),(63.66,0.001823),(66.08,0.001428),(68.52,0.001076),(70.99,0.000775),(73.49,0.000523),(76,0.000326))

DEATH RATES TABLE[MALE, AG 30 TO 34]([(0,0)-(80,0.02)],(0,1),(19.92,0.017569),(22.3,0.016098),(24.66,0.014776),(27.01,0.013578),(29.32,0.012486),(31.64,0.011483),(33.95,0.010557),(36.23,0.009698),(38.5,0.008899),(40.63,0.008222),(42.86,0.007568),(45.12,0.006872),(47.37,0.006215),(49.62,0.005592),(51.87,0.005002),(54.11,0.004443),(56.34,0.003915),(58.58,0.003409),(61.25,0.002798),(63.66,0.002297),(66.08,0.001851),(68.52,0.001442),(70.99,0.001078),(73.49,0.000763),(76,0.000503))

DEATH RATES TABLE[MALE, AG 35 TO 39]([(0,0)-(80,0.02)],(0,1),(19.92,0.019279),(22.3,0.017682),(24.66,0.016247),(27.01,0.014948),(29.32,0.013764),(31.64,0.012675),(33.95,0.011671),(36.23,0.010742),(38.5,0.009877),(40.63,0.009146),(42.86,0.00845),(45.12,0.007706),(47.37,0.006999),(49.62,0.006328),(51.87,0.00569),(54.11,0.005083),(56.34,0.004508),(58.58,0.003959),(61.25,0.00329),(63.66,0.002741),(66.08,0.002236),(68.52,0.001766),(70.99,0.001342),(73.49,0.000968),(76,0.000651))

DEATH RATES TABLE[MALE, AG 40 TO 44]([(0,0)-(80,0.04)],(0,1),(19.92,0.022776),(22.3,0.02095),(24.66,0.019312),(27.01,0.017831),(29.32,0.01648),(31.64,0.015242),(33.95,0.014098),(36.23,0.013042),(38.5,0.012057),(40.63,0.011225),(42.86,0.010446),(45.12,0.009623),(47.37,0.008833),(49.62,0.008075),(51.87,0.007349),(54.11,0.006656),(56.34,0.005995),(58.58,0.005361),(61.25,0.004582),(63.66,0.00391),(66.08,0.003273),(68.52,0.002666),(70.99,0.002097),(73.49,0.001577),(76,0.001116))

DEATH RATES TABLE[MALE, AG 45 TO 49]([(0,0)-(80,0.04)],(0,1),(19.92,0.026511),(22.3,0.024484),(24.66,0.022667),(27.01,0.021024),(29.32,0.019528),(31.64,0.018155),(33.95,0.016891),(36.23,0.015723),(38.5,0.014634),(40.63,0.013715),(42.86,0.012852),(45.12,0.011973),(47.37,0.011121),(49.62,0.010298),(51.87,0.009503),(54.11,0.008737),(56.34,0.008001),(58.58,0.00729),(61.25,0.006434),(63.66,0.005643),(66.08,0.004861),(68.52,0.004095),(70.99,0.00335),(73.49,0.00264),(76,0.00198))

DEATH RATES TABLE[MALE, AG 50 TO 54]([(0,0)-(80,0.04)],(0,1),(19.92,0.033434),(22.3,0.031013),(24.66,0.028845),(27.01,0.026888),(29.32,0.025108),(31.64,0.023477),(33.95,0.021977),(36.23,0.020588),(38.5,0.019299),(40.63,0.018179),(42.86,0.017155),(45.12,0.016154),(47.37,0.015174),(49.62,0.014219),(51.87,0.013291),(54.11,0.012391),(56.34,0.01152),(58.58,0.010673),(61.25,0.009678),(63.66,0.008686),(66.08,0.007685),(68.52,0.006675),(70.99,0.00566),(73.49,0.004655),(76,0.003675))

DEATH RATES TABLE[MALE, AG 55 TO 59]([(0,0)-(80,0.06)],(0,1),(19.92,0.044138),(22.3,0.04106),(24.66,0.038314),(27.01,0.035841),(29.32,0.033597),(31.64,0.031544),(33.95,0.02966),(36.23,0.027918),(38.5,0.026304),(40.63,0.024908),(42.86,0.023655),(45.12,0.022423),(47.37,0.021217),(49.62,0.020035),(51.87,0.018882),(54.11,0.017758),(56.34,0.016668),(58.58,0.015606),(61.25,0.014384),(63.66,0.013094),(66.08,0.011774),(68.52,0.010417),(70.99,0.00903),(73.49,0.007621),(76,0.006211))

DEATH RATES TABLE[MALE, AG 60 TO 64]([(0,0)-(80,0.08)],(0,1),(19.92,0.065496),(22.3,0.060868),(24.66,0.056767),(27.01,0.053092),(29.32,0.049774),(31.64,0.046754),(33.95,0.04399),(36.23,0.041446),(38.5,0.039096),(40.63,0.037084),(42.86,0.035279),(45.12,0.033503),(47.37,0.031763),(49.62,0.03006),(51.87,0.0284),(54.11,0.026783),(56.34,0.025216),(58.58,0.023688),(61.25,0.0219),(63.66,0.020022),(66.08,0.018091),(68.52,0.0161),(70.99,0.014056),(73.49,0.011965),(76,0.009852))

DEATH RATES TABLE[MALE, AG 65 TO 69]([(0,0)-(80,0.1)],(0,1),(19.92,0.096446),(22.3,0.089546),(24.66,0.083489),(27.01,0.07811),(29.32,0.073284),(31.64,0.068925),(33.95,0.064956),(36.23,0.061321),(38.5,0.057978),(40.63,0.055173),(42.86,0.052635),(45.12,0.05014),(47.37,0.047696),(49.62,0.045308),(51.87,0.042977),(54.11,0.040713),(56.34,0.038515),(58.58,0.036376),(61.25,0.033787),(63.66,0.031099),(66.08,0.028317),(68.52,0.025433),(70.99,0.022445),(73.49,0.019361),(76,0.016207))

DEATH RATES TABLE[MALE, AG 70 TO 74]([(0,0)-(80,0.2)],(0,1),(19.92,0.149635),(22.3,0.138669),(24.66,0.129223),(27.01,0.120967),(29.32,0.113663),(31.64,0.107132),(33.95,0.10125),(36.23,0.09591),(38.5,0.091032),(40.63,0.086976),(42.86,0.083426),(45.12,0.079866),(47.37,0.07638),(49.62,0.072976),(51.87,0.069655),(54.11,0.066431),(56.34,0.063306),(58.58,0.060262),(61.25,0.056487),(63.66,0.052506),(66.08,0.048388),(68.52,0.044078),(70.99,0.039558),(73.49,0.034824),(76,0.02989))

DEATH RATES TABLE[MALE, AG 75 TO 79]([(0,0)-(80,0.4)],(0,1),(19.92,0.22951),(22.3,0.212135),(24.66,0.197665),(27.01,0.185337),(29.32,0.174656),(31.64,0.165277),(33.95,0.156946),(36.23,0.149482),(38.5,0.142737),(40.63,0.137144),(42.86,0.132341),(45.12,0.127625),(47.37,0.123009),(49.62,0.118498),(51.87,0.114104),(54.11,0.109833),(56.34,0.105693),(58.58,0.101661),(61.25,0.096657),(63.66,0.091211),(66.08,0.085513),(68.52,0.079468),(70.99,0.073022),(73.49,0.066131),(76,0.058764))

DEATH RATES TABLE[MALE, AG 80 AND OVER]([(0,0)-(80,0.4)],(0,1),(19.92,0.387521),(22.3,0.371088),(24.66,0.356328),(27.01,0.34296),(29.32,0.330768),(31.64,0.319573),(33.95,0.309246),(36.23,0.299675),(38.5,0.290759),(40.63,0.28317),(42.86,0.2765),(45.12,0.269829),(47.37,0.263169),(49.62,0.256538),(51.87,0.249959),(54.11,0.243444),(56.34,0.237022),(58.58,0.23066),(61.25,0.222615),(63.66,0.213667),(66.08,0.204084),(68.52,0.193666),(70.99,0.182251),(73.49,0.16969),(76,0.155821))

Units: Dmnl/Year

deaths[sex,age]= (Population[sex,age] * non malaria death rates[sex,age])+Malaria Deaths[sex,age]

Units: Person/Year

deaths per age group[sex,AG UNDER 1]= deaths[sex,AGE 0]

deaths per age group[sex,AG 1 TO 4]= SUM(deaths[sex,age 1 to 4!])

deaths per age group[sex,AG 5 TO 9]= SUM(deaths[sex,age 5 to 9!])

deaths per age group[sex,AG 10 TO 14]=SUM(deaths[sex,age 10 to 14!])

deaths per age group[sex,AG 15 TO 19]=SUM(deaths[sex,age 15 to 19!])

deaths per age group[sex,AG 20 TO 24]=SUM(deaths[sex,age 20 to 24!])

deaths per age group[sex,AG 25 TO 29]=SUM(deaths[sex,age 25 to 29!])

deaths per age group[sex,AG 30 TO 34]=SUM(deaths[sex,age 30 to 34!])

deaths per age group[sex,AG 35 TO 39]=SUM(deaths[sex,age 35 to 39!])

deaths per age group[sex,AG 40 TO 44]=SUM(deaths[sex,age 40 to 44!])

deaths per age group[sex,AG 45 TO 49]=SUM(deaths[sex,age 45 to 49!])

deaths per age group[sex,AG 50 TO 54]=SUM(deaths[sex,age 50 to 54!])

deaths per age group[sex,AG 55 TO 59]=SUM(deaths[sex,age 55 to 59!])

deaths per age group[sex,AG 60 TO 64]=SUM(deaths[sex,age 60 to 64!])

deaths per age group[sex,AG 65 TO 69]=SUM(deaths[sex,age 65 to 69!])

deaths per age group[sex,AG 70 TO 74]=SUM(deaths[sex,age 70 to 74!])

deaths per age group[sex,AG 75 TO 79]=SUM(deaths[sex,age 75 to 79!])

deaths per age group[sex,AG 80 AND OVER]=

deaths[sex,AGE 80 AND OVER]

Units: Person/Year

desired fertility rate= INITIAL DESIRED FERTILITY RATE*Effect Of Income On Desired Fertility

Units: Dmnl

Effect Of Education On Contraceptive Prevalence= SMOOTH N(relative average years schooling^ELASTICITY OF CONTRACEPTIVE PREVALENCE TO YEARS OF SCHOOLING, TIME FOR EDUCATION TO AFFECT CONTRACEPTIVE PREVALENCE, relative average years schooling^ELASTICITY OF CONTRACEPTIVE PREVALENCE TO YEARS OF SCHOOLING, 1)

Units: Dmnl

Effect Of Income On Desired Fertility= SMOOTH N(relative real pc GDP^ELASTICITY OF DESIRED FERTILITY RATE TO INCOME, TIME FOR INCOME TO AFFECT DESIRED FERTILITY, relative real pc GDP^ELASTICITY OF DESIRED FERTILITY RATE TO INCOME,1)

Units: Dmnl

effective death rates per age group[sex, age group]= MIN(1,deaths per age group[sex,age group]/population group[sex,age group])

Units: Dmnl/Year

ELASTICITY OF CONTRACEPTIVE PREVALENCE TO YEARS OF SCHOOLING= 1.5

Units: Dmnl

ELASTICITY OF DESIRED FERTILITY RATE TO INCOME= -0.4

Units: Dmnl

FERTILITY DELAY= 1

Units: Year

indicated age specific fertility distribution[ag childbearing]= AGE SPECIFIC FERTILITY DISTRIBUTION TABLE(ag childbearing-1-FERTILITY DELAY)

Units: Dmnl/Year

indicated migrants age distribution[age]= MIGRANTS AGE DISTRIBUTION TABLE(age-1)

Units: Dmnl

INITIAL CONTRACEPTIVE PREVALENCE= 0.117

Units: Dmnl

INITIAL DESIRED FERTILITY RATE= 4

Units: Dmnl

INITIAL population[sex, age]

Units: Person

life expectancy[sex]= SUM(measured life expectancy[sex,age group!]*age group weight[sex,age group!])

Units: Year

measured life expectancy [sex, age group]= LOOKUP INVERT(DEATH RATES TABLE[sex,age group], effective death rates per age group[sex,age group])

Units: Year

migrants age distribution[age]= indicated migrants age distribution[age]/SUM(indicated migrants age distribution[age!])

Units: Dmnl

MIGRANTS AGE DISTRIBUTION TABLE([(0,0)-(80,0.2)],(0,0),(5,0),(10,0),(15,0.1),(20,0.12),(25,0.13),(30,0.13),(35,0.12),(40,0.1),(45,0.08),(50,0.05),(55,0.025),(60,0),(65,0),(70,0),(75,0),(80,0))

Units: Dmnl

MIGRANTS SEX DISTRIBUTION[sex]= 0.6, 0.4

Units: Dmnl

migration[sex, age]= net total migration*MIGRANTS SEX DISTRIBUTION[sex]*migrants age distribution[age]

Units: Person/Year

NATURAL FERTILITY RATE= 7.25

Units: Dmnl

NET migration per thousands habitants

Units: Dmnl/Year

net total migration= (SUM(Population[sex!,age!])/1000)*NET migration per thousands habitants

Units: Person/Year

population group[sex,AG UNDER 1]=

Population[sex,AGE 0]

population group[sex,AG 1 TO 4]=

SUM(Population[sex,age 1 to 4!])

population group[sex,AG 5 TO 9]=SUM(Population[sex,age 5 to 9!])

population group[sex,AG 10 TO 14]=SUM(Population[sex,age 10 to 14!])

population group[sex,AG 15 TO 19]=SUM(Population[sex,age 15 to 19!])

population group[sex,AG 20 TO 24]=SUM(Population[sex,age 20 to 24!])

population group[sex,AG 25 TO 29]=SUM(Population[sex,age 25 to 29!])

population group[sex,AG 30 TO 34]=SUM(Population[sex,age 30 to 34!])

population group[sex,AG 35 TO 39]=SUM(Population[sex,age 35 to 39!])

population group[sex,AG 40 TO 44]=SUM(Population[sex,age 40 to 44!])

population group[sex,AG 45 TO 49]=SUM(Population[sex,age 45 to 49!])

population group[sex,AG 50 TO 54]=SUM(Population[sex,age 50 to 54!])

population group[sex,AG 55 TO 59]=SUM(Population[sex,age 55 to 59!])

population group[sex,AG 60 TO 64]=SUM(Population[sex,age 60 to 64!])

population group[sex,AG 65 TO 69]=SUM(Population[sex,age 65 to 69!])

population group[sex,AG 70 TO 74]=SUM(Population[sex,age 70 to 74!])

population group[sex,AG 75 TO 79]=SUM(Population[sex,age 75 to 79!])

population group[sex,AG 80 AND OVER]=Population[sex,AGE 80 AND OVER]

Units: Person

population under five[sex]= population group[sex,AG UNDER 1]+ population group[sex,AG 1 TO 4]

Units: Person

proportion of babies by sex[FEMALE]= 0.4926

proportion of babies by sex[MALE]= 1-proportion of babies by sex[FEMALE]

Units: Dmnl

REFERENCE CUMULATIVE FERTILITY DISTRIBUTION= 1

Units: Dmnl/Year

sexually active women[childbearing age]= Population[FEMALE,childbearing age]

Units: Person

share of population per gender[FEMALE]= SUM(Population[FEMALE, age!])/SUM(Population[sex!, age!])

share of population per gender [MALE]= 1-share of population per gender[FEMALE]

Units: Dmnl

TIME FOR EDUCATION TO AFFECT CONTRACEPTIVE PREVALENCE= 10

Units: Year

TIME FOR INCOME TO AFFECT DESIRED FERTILITY= 1

Units: Year

total births= SUM(births[sex!])

Units: Person/Year

total deaths= SUM(total deaths by sex[sex!])

Units: Person/Year

total deaths by sex[sex]= SUM(deaths[sex,age!])

Units: Person/Year

total fertility rate= desired fertility rate*contraceptive prevalence+NATURAL FERTILITY RATE*(1-contraceptive prevalence)

Units: Dmnl

********************************

2 Education

********************************

ADJUSTMENT TIME FOR AVERAGE YEARS OF SCHOOLING= 30

Units: Year

average adult literacy rate= 100-100/((Average Years Of Schooling*MULTIPLIER+1)^EXPONENT)

Units: Dmnl

Average Years Of Schooling= INTEG (change in average years of schooling, INITIAL AVERAGE YEARS OF SCHOOLING)

Units: Year

change in average years of schooling= (target years of schooling-Average Years Of Schooling)/ADJUSTMENT TIME FOR AVERAGE YEARS OF SCHOOLING

Units: Dmnl

education expenditure= real GDP*EDUCATION expenditure as a fraction of GDP

Units: $/Year

EDUCATION expenditure as a fraction of GDP

Units: Dmnl

education expenditure per pupil= education expenditure/SUM(population of school going age[sex!])

Units: $/Year/Person

ELASTICITY OF TARGET YEARS OF SCHOOLING TO EDUCATION EXPENDITURE= 0.175

Units: Dmnl

EXPONENT= 155

Units: Dmnl

INITIAL AVERAGE YEARS OF SCHOOLING= 0.825

Units: Year

INITIAL EDUCATION EXPENDITURE PER PUPIL= INITIAL(education expenditure per pupil)

Units: $/Year/Person

INITIAL TARGET YEARS OF SCHOOLING= 4.1

Units: Year

MULTIPLIER= 0.002

Units: 1/Year

population of school going age[sex]= SUM(Population[sex,school age!])

Units: Person

relative education expenditure per pupil= education expenditure per pupil/INITIAL EDUCATION EXPENDITURE PER PUPIL

Units: Dmnl

target years of schooling= INITIAL TARGET YEARS OF SCHOOLING*relative education expenditure per pupil^ELASTICITY OF TARGET YEARS OF SCHOOLING TO EDUCATION EXPENDITURE

Units: Year

********************************

3 Health

********************************

health expenditure=real GDP*HEALTH expenditure as a fraction of GDP

Units: $/Year

HEALTH expenditure as a fraction of GDP

Units: Dmnl

INCOME WEIGHT= 0.75

Units: Dmnl

indicated average life expectancy at birth= LEFT SATURATION POINT+(RIGHT SATURATION POINT-LEFT SATURATION POINT)*(1/(EXP(-STEEPNESS*well being index)*EXP(INFLECTION POINT*STEEPNESS)+1)-(EXP(-STEEPNESS*well being index)/(EXP(INFLECTION POINT*STEEPNESS)+1)))

Units: Year

indicated life expectancy at birth[FEMALE]= (indicated average life expectancy at birth+MALE FEMALE LIFE EXPECTANCY DIFFERENCE/2)

indicated life expectancy at birth[MALE]=(indicated life expectancy at birth[FEMALE]-MALE FEMALE LIFE EXPECTANCY DIFFERENCE)

Units: Year

INFLECTION POINT= -0.01986

Units: Dmnl

LEFT SATURATION POINT= 29.5

Units: Year

MALE FEMALE LIFE EXPECTANCY DIFFERENCE= 4

Units: Year

pc health expenditure= health expenditure/total population

Units: $/Year/Person

Perceived Real Pc GDP= SMOOTH N(real pc GDP,TIME FOR INCOME CHANGES TO AFFECT LIFE EXPECTANCY,540,1)

Units: $/(Year*Person)

REFERENCE SATURATION INCOME= 25000

Units: $/(Year*Person)

REFERENCE SATURATION PC HEALTH EXPENDITURE= 300

Units: $/(Person*Year)

RIGHT SATURATION POINT= 82.2

Units: Year

STEEPNESS= 17

Units: Dmnl [0,60]

TIME FOR INCOME CHANGES TO AFFECT LIFE EXPECTANCY= 10

Units: Year

well being index= (Perceived Real Pc GDP/REFERENCE SATURATION INCOME)*INCOME WEIGHT+ (Effective Pc Health Expenditure/REFERENCE SATURATION PC HEALTH EXPENDITURE)*(1-INCOME WEIGHT)

Units: Dmnl

********************************

4 Production

********************************

agricultural export outside SSA= real GDP*AGRICULTURAL exports to outside SSA as share of GDP

Units: $/Year

AGRICULTURAL exports to outside SSA as share of GDP

Units: Dmnl

AVERAGE DEPRECIATION TIME= 20

Units: Year

average life expectancy= life expectancy[FEMALE]*share of population per gender[FEMALE]+life expectancy[MALE]*share of population per gender[MALE]

Units: Year

Capital= INTEG (gross capital formation-depreciation, INITIAL CAPITAL)

Units: $

depreciation= Capital/AVERAGE DEPRECIATION TIME

Units: $/Year

effect of life expectancy on TFP= relative life expectancy^ELASTICITY OF TFP TO LIFE EXPECTANCY

Units: Dmnl

effect of years of schooling on TFP= relative average years schooling^ELASTICITY OF TFP TO YEARS OF SCHOOLING

Units: Dmnl

ELASTICITY OF PROPENSITY TO SAVE TO INCOME= 0.5

Units: Dmnl

ELASTICITY OF TFP TO LIFE EXPECTANCY= 0.5

Units: Dmnl

ELASTICITY OF TFP TO YEARS OF SCHOOLING= 0.115

Units: Dmnl

gross capital formation= investment

Units: $/Year

INITIAL AVERAGE LIFE EXPECTANCY= INITIAL(average life expectancy)

Units: Year

INITIAL CAPITAL= 7.5e+011

Units: $

INITIAL LABOR FORCE= INITIAL(labor force)

Units: Person

INITIAL PRODUCTION= 1.58585e+011

Units: $/Year

INITIAL PROPENSITY TO SAVE= 0.2

Units: Dmnl

INITIAL REAL GDP GROWTH RATE= 0.055

Units: Dmnl/Year

INITIAL REAL PC GDP= INITIAL(real pc GDP)

Units: $/(Year*Person)

investment= real GDP*propensity to save

Units: $/Year

propensity to consume= 1-propensity to save

Units: Dmnl

propensity to save= MIN(1, INITIAL PROPENSITY TO SAVE*relative real pc GDP^ELASTICITY OF PROPENSITY TO SAVE TO INCOME)

Units: Dmnl

real GDP growth rate= TREND(real GDP, TIME HORIZON TO MEASURE GROWTH RATE, INITIAL REAL GDP GROWTH RATE)

Units: Dmnl/Year

real pc GDP= real GDP/total population

Units: $/(Year*Person)

relative capital= Capital/INITIAL CAPITAL

Units: Dmnl

relative labor force= labor force/INITIAL LABOR FORCE

Units: Dmnl

relative life expectancy= average life expectancy/INITIAL AVERAGE LIFE EXPECTANCY

Units: Dmnl

relative production= (relative capital^CAPITAL SHARE)*(relative labor force^(1-CAPITAL SHARE))*total factor productivity

Units: Dmnl

relative real pc GDP= real pc GDP/INITIAL REAL PC GDP

Units: Dmnl

TIME HORIZON TO MEASURE GROWTH RATE= 1

Units: Year

total factor productivity= effect of life expectancy on TFP*effect of years of schooling on TFP*effect of malaria prevalence on productivity

Units: Dmnl

********************************

5 Malaria transmission

********************************

area fraction covered by environmental management= total area covered by environmental management/area of population living in malaria risk areas

Units: Dmnl

area of population living in malaria risk areas= TOTAL LAND AREA OF MALARIA ENDEMIC COUNTRIES*proportion of population living in malaria risk areas

Units: Square kilometer

AVERAGE DURATION OF IMMUNITY AND INFECTIVENESS= 0.5

Units: Year

CLIMATE suitability index[region]

Units: Dmnl

deaths due to other causes[sex, age]= Malaria Infectious And Partially Immune Population[sex,age]*non malaria death rates[sex,age]

Units: Person/Year

EFFECT OF EDUCATION LEVEL ON PROTECTIVE MEASURES EFFICACY([(0,0)-(9,1)],(0,0.25),(9,1))

Units: Dmnl

effectiveness of protective measures= EFFECT OF EDUCATION LEVEL ON PROTECTIVE MEASURES EFFICACY(relative average years schooling)

Units: Dmnl

EM coverage= POPULATION DENSITY TABLE(area fraction covered by environmental management)*PROPORTIONAL REDUCTION IN RISK FOR EM COVERED POPULATION

Units: Dmnl

indicated protective measures coverage fraction= (bednet covered population+Population Covered By Non Bednets Protective Measures)/total population living in malaria risk areas

Units: Dmnl

infected recovered[sex,age]= Malaria Infectious And Partially Immune Population[sex,age]/(AVERAGE DURATION OF IMMUNITY AND INFECTIVENESS*effect of treatment coverage on malaria mortality)

Units: Person/Year

INITIAL MALARIA INFECTIOUS AND PARTIALLY IMMUNE FRACTION= 0.25

Units: Dmnl

integrated vector management coverage fraction= MIN(1,MAX(EM coverage, MAX(IRS coverage,protective measures coverage))+(total indicated IVM coverage-MAX(EM coverage,MAX(IRS coverage,protective measures coverage)))*(1-INTERVENTIONS OVERLAPPING FACTOR))

Units: Dmnl

IRS coverage= total proportion of population covered by IRS*PROPORTIONAL REDUCTION IN RISK FOR IRS COVERED POPULATION

Units: Dmnl

malaria cases[sex,age]= Malaria Infectious And Partially Immune Population[sex,age]*MALARIA CONTACT RATE*MALARIA INFECTIVITY*(non infected vulnerable population[sex,age]/vulnerable population[sex,age])

Units: Person/Year

MALARIA CONTACT RATE= 3.65

Units: Dmnl/Year

Malaria Deaths[sex,age]= DELAY N(malaria cases[sex,age]*malaria fractional mortality rate[age], TIME STEP, malaria cases[sex,age]*malaria fractional mortality rate[age],1)

Units: Person/Year

malaria deaths among under five= SUM(Malaria Deaths[sex!,age under five!])

Units: Person/Year

malaria deaths among under five as share of total deaths= IF THEN ELSE(total malaria deaths<10, 0, malaria deaths among under five/total malaria deaths)

Units: Dmnl

malaria fractional mortality rate[age]= NATURAL MALARIA FRACTIONAL MORTALITY RATE[age]*effect of treatment coverage on malaria mortality

Units: Dmnl

MALARIA INFECTIVITY= 1

Units: Dmnl

NATURAL MALARIA FRACTIONAL MORTALITY RATE[age under five]= 0.025

NATURAL MALARIA FRACTIONAL MORTALITY RATE[age five and over]= 0.001

Units: Dmnl

non infected vulnerable population[sex,age]= MAX(0,vulnerable population[sex,age]-Malaria Infectious And Partially Immune Population[sex,age])

Units: Person

non malaria death rates[sex,age]= MIN(1,MAX(0,DEATH RATES TABLE[sex,age group](indicated life expectancy at birth[sex])))

Units: Dmnl/Year

POPULATION DENSITY TABLE([(0,0)-(1,1)],(0,0),(1.19e-006,0.000588),(3.26e-006,0.00153),(4.58e-006,0.00212),(7.12e-006,0.00314),(8.44e-006,0.00366),(1.07e-005,0.00454),(1.24e-005,0.00516),(1.48e-005,0.00601),(3.95e-005,0.0147),(4.83e-005,0.0177),(5.76e-005,0.0209),(6.73e-005,0.0242),(7.14e-005,0.0255),(8.51e-005,0.0298),(8.75e-005,0.0305),(9.11e-005,0.0315),(9.34e-005,0.0321),(9.44e-005,0.0324),(9.79e-005,0.0333),(0.000103,0.0346),(0.000105,0.0351),(0.000112,0.0368),(0.000115,0.0377),(0.000119,0.0386),(0.000126,0.0402),(0.000134,0.0421),(0.000137,0.0428),(0.000179,0.0526),(0.000184,0.0535),(0.000204,0.0582),(0.000207,0.0588),(0.000216,0.0604),(0.000223,0.0619),(0.000233,0.0637),(0.00024,0.0652),(0.000241,0.0652),(0.000261,0.0687),(0.000267,0.0699),(0.000277,0.0714),(0.00029,0.0736),(0.000305,0.076),(0.000308,0.0765),(0.000311,0.0769),(0.000319,0.0782),(0.000323,0.0789),(0.000327,0.0794),(0.000361,0.0847),(0.000365,0.0854),(0.000383,0.088),(0.000387,0.0885),(0.000396,0.0898),(0.000403,0.0907),(0.000406,0.0911),(0.000409,0.0915),(0.000412,0.0918),(0.000426,0.0935),(0.000431,0.0941),(0.00046,0.0974),(0.000466,0.098),(0.000482,0.0996),(0.000487,0.1),(0.000526,0.104),(0.000561,0.107),(0.000567,0.108),(0.000573,0.108),(0.000598,0.111),(0.000619,0.112),(0.000627,0.113),(0.000734,0.121),(0.000735,0.121),(0.000738,0.121),(0.000748,0.121),(0.000751,0.122),(0.000761,0.122),(0.000779,0.123),(0.000828,0.126),(0.000839,0.126),(0.00086,0.127),(0.00087,0.127),(0.001,0.133),(0.002,0.163),(0.003,0.183),(0.004,0.199),(0.005,0.213),(0.006,0.225),(0.007,0.235),(0.008,0.244),(0.009,0.253),(0.01,0.261),(0.02,0.319),(0.03,0.359),(0.04,0.391),(0.05,0.417),(0.1,0.511),(0.15,0.575),(0.2,0.625),(0.25,0.667),(0.3,0.704),(0.35,0.736),(0.4,0.765),(0.45,0.792),(0.5,0.817),(0.55,0.84),(0.6,0.861),(0.65,0.882),(0.7,0.901),(0.75,0.919),(0.8,0.937),(0.85,0.954),(0.9,0.97),(0.95,0.985),(1,1))

Units: Dmnl

population living in malaria risk areas by region[sex,age,region]= population by region[sex,age,region]*CLIMATE suitability index[region]

Units: Person

PROPORTION OF DEATHS REPORTED= 0.1

Units: Dmnl

PROPORTION of population by region[region]

Units: Dmnl

proportion of population living in malaria risk areas= total population living in malaria risk areas/total population

Units: Dmnl

protective measures coverage= effectiveness of protective measures*indicated protective measures coverage fraction

Units: Dmnl

reported malaria deaths[sex, age]= Malaria Deaths[sex,age]*PROPORTION OF DEATHS REPORTED

Units: Person/Year

REPORTED PROPORTION OF MALARIA CASES= 0.1

Units: Dmnl

total indicated IVM coverage= EM coverage+IRS coverage+protective measures coverage

Units: Dmnl

TOTAL LAND AREA OF MALARIA ENDEMIC COUNTRIES= 2.36142e+007

Units: Square kilometer

total malaria deaths= SUM(Malaria Deaths[sex!,age!])

Units: Person/Year

total reported malaria cases= SUM(malaria cases[sex!,age!])*REPORTED PROPORTION OF MALARIA CASES

Units: Person/Year

total reported malaria deaths= SUM(reported malaria deaths[sex!,age!])

Units: Person/Year

under five malaria cases= SUM(malaria cases[sex!,age under five!])

Units: Person/Year

********************************

6 Malaria DDT

********************************

AIRBORNE FRACTION= 0.4

Units: Dmnl

application rate to air= AIRBORNE FRACTION*crop application rate

Units: Ton/Year

application rate to soil= (1-AIRBORNE FRACTION)*crop application rate

Units: Ton/Year

BODY WEIGHTS EATEN PER YEAR= 10

Units: Dmnl/Year

CONSUMED FRACTION= 0.5

Units: Dmnl

covered land= crop application rate/CROP APPLICATION

Units: Square kilometer

CROP APPLICATION= 0.1122

Units: Ton/(Square kilometer*Year)

crop application rate= DDT application rate-DDT application rate to dwellings

Units: Ton/Year

DDT application rate

Units: Ton/Year

DDT application rate to dwellings= Population Covered By DDT Application*DDT REQUIRED QUANTITY PER PERSON PER YEAR/KG PER TON

Units: Ton/Year

DDT concentration in fish= DDT In Fish/MASS OF FISH

Units: Dmnl

DDT concentration in ocean= DDT In Oceans/MASS OF MIXED LAYER

Units: Dmnl

DDT concentration in pankton= DDT concentration in ocean*"OCEAN-PANKTON CONCENTRATION FACTOR"

Units: Dmnl

DDT In Air= INTEG (application rate to air+evaporation rate from dwelling+evaporation rate from soil-precipitation rate into oceans-precipitation rate to soil,14645)

Units: Ton

DDT In Fish= INTEG (uptake rate in fish-dead fish remaining in oceans-fish consumed-harmless excretion rate-toxic excretion rate, 227)

Units: Ton

DDT In Oceans= INTEG (dead fish remaining in oceans+precipitation rate into oceans+"run-off rate"+toxic excretion rate-degradation rate in oceans-uptake rate in fish, 1.419e+006)

Units: Ton

DDT In Rivers= INTEG (solution rate-"run-off rate", 68.32)

Units: Ton

DDT In Soil= INTEG (application rate to soil+precipitation rate to soil-degradation rate in soil-evaporation rate from soil-HARVESTING-solution rate, 342031)

Units: Ton

DDT production

Units: Ton/Year

DDT REQUIRED QUANTITY PER PERSON PER YEAR= 0.2

Units: Kg/Person/Year

dead fish remaining in oceans= (1-CONSUMED FRACTION)*deaths of fish

Units: Ton/Year

deaths of fish= DDT In Fish/(1.5*HALF LIFE OF FISH)

Units: Ton/Year

DEGRADATION HALF LIFE IN OCEAN= 15

Units: Year

DEGRADATION HALF LIFE IN SOIL= 10.5

Units: Year

degradation rate in oceans= DDT In Oceans/(1.44*DEGRADATION HALF LIFE IN OCEAN)

Units: Ton/Year

degradation rate in soil= DDT In Soil/(1.44*DEGRADATION HALF LIFE IN SOIL)

Units: Ton/Year

degradation rate on dwellings= DDT On Dwellings/(1.44*DEGRADATION HALF LIFE IN SOIL)

Units: Ton/Year

DEGRADED FRACTION= 0.1

Units: Dmnl

EVAPORATION HALF LIFE IN SOIL= 2

Units: Year

evaporation rate from dwelling= DDT On Dwellings/(1.44*EVAPORATION HALF LIFE IN SOIL)

Units: Ton/Year

evaporation rate from soil= DDT In Soil/(1.44*EVAPORATION HALF LIFE IN SOIL)

Units: Ton/Year

EXCRETION HALF LIFE= 0.3

Units: Year

excretion rate from fish= DDT In Fish/(1.5*EXCRETION HALF LIFE)

Units: Ton/Year

fish consumed= CONSUMED FRACTION*deaths of fish

Units: Ton/Year

HALF LIFE OF FISH= 3

Units: Year

harmless excretion rate= DEGRADED FRACTION*excretion rate from fish

Units: Ton/Year

HARVESTING= 100

Units: Ton/Year

MASS OF FISH= 6e+008

Units: Ton

MASS OF MIXED LAYER= 3e+016

Units: Ton

"OCEAN-PANKTON CONCENTRATION FACTOR"= 2000

Units: Dmnl

PRECIPITATION HALF LIFE= 0.05

Units: Year

precipitation rate into oceans= (1-SOIL FRACTION)*DDT In Air/(1.44*PRECIPITATION HALF LIFE)

Units: Ton/Year

precipitation rate to soil= SOIL FRACTION*DDT In Air/(1.44*PRECIPITATION HALF LIFE)

Units: Ton/Year

"RUN-OFF HALF LIFE"= 0.1

Units: Year

"run-off rate"= DDT In Rivers/(1.44*"RUN-OFF HALF LIFE")

Units: Ton/Year

SOIL FRACTION= 0.3

Units: Dmnl

SOLUTION HALF LIFE= 500

Units: Year

solution rate= DDT In Soil/(1.44*SOLUTION HALF LIFE)

Units: Ton/Year

Stocked DDT= INTEG (DDT production-DDT application rate, 175000)

Units: Ton

toxic excretion rate= (1-DEGRADED FRACTION)*excretion rate from fish

Units: Ton/Year

uptake rate in fish= BODY WEIGHTS EATEN PER YEAR*MASS OF FISH*DDT concentration in pankton

Units: Ton/Year

********************************

7 Malaria IVM interventions

********************************

Area Covered By EM= INTEG (Treating Areas With EM-EM loss of efficacy, INITIAL AREA COVERED BY EM)

Units: Square kilometer

area covered by larviciding= larviciding expenditure/LARVICIDING UNIT COST

Units: Square kilometer

bednet covered population= Bednets In Households*COVERAGE PER BEDNET

Units: Person

Bednets In Households= INTEG (bednets distributed-discarded bednets, INITIAL BEDNETS IN HOUSEHOLDS)

Units: Bednet

budget for bednets= bednet expenditure*FRACTION OF PROTECTIVE MEASURES EXPENDITURE FOR BEDNETS

Units: $/Year

budget for non bednets protective measures= bednet expenditure*(1-FRACTION OF PROTECTIVE MEASURES EXPENDITURE FOR BEDNETS)

Units: $/Year

DDT efficacy= MAX DDT EFFICACY-proportion of population covered by DDT*MAX DDT EFFICACY

Units: Dmnl

DDT expenditure= IRS expenditure*IF THEN ELSE(Time < 2012, SHARE of IRS budget for DDT, FUTURE SHARE OF IRS BUDGET FOR DDT)

Units: $/Year

discarded bednets= Bednets In Households/AVERAGE BEDNET EFFECTIVE DURATION

Units: Bednet/Year

EM expenditure for infrastructure and household modifications= EM expenditure*SHARE OF EM EXPENDITURE FOR INFRASTRUCTURE AND HOUSEHOLD MODIFICATIONS TIME SERIES(Time)

Units: $/Year

EM loss of efficacy= Area Covered By EM/AVERAGE DURATION OF EFFECTIVENESS OF EM INTERVENTIONS

Units: Square kilometer/Year

fraction of chemical control expenditure for non DDT IRS= 1-IF THEN ELSE(Time < 2012, SHARE of IRS budget for DDT, FUTURE SHARE OF IRS BUDGET FOR DDT)

Units: Dmnl

FRACTION OF PROTECTIVE MEASURES EXPENDITURE FOR BEDNETS= 1

Units: Dmnl

INITIAL AREA COVERED BY EM= 1

Units: Square kilometer

INITIAL BEDNETS IN HOUSEHOLDS= 0

Units: Bednet

larviciding expenditure= EM expenditure-EM expenditure for infrastructure and household modifications

Units: $/Year

NON BEDNETS PROTECTIVE MEASURES PRICE PER PERSON PER YEAR= 20

Units: $/(Year*Person)

non DDT efficacy= MAX NON DDT EFFICACY-proportion of population covered by non DDT IRS*MAX NON DDT EFFICACY

Units: Dmnl

Population Covered By Non Bednets Protective Measures= DELAY N(budget for non bednets protective measures/NON BEDNETS PROTECTIVE MEASURES PRICE PER PERSON PER YEAR, TIME FOR NON BEDNET PROTECTIVE MEASURES IMPLEMENTATION,0,1)

Units: Person

Population Covered By Non DDT IRS Application= DELAY N(non DDT IRS expenditure/NON DDT IRS UNIT COST, TIME FOR IRS DELPOYMENT,1.8e+006,1)

Units: Person

population effectively covered by DDT= proportion of population covered by DDT*(DDT efficacy+(MAX DDT EFFICACY-DDT efficacy)/2)

Units: Dmnl

population effectively covered by non DDT IRS= proportion of population covered by non DDT IRS*(non DDT efficacy+(MAX NON DDT EFFICACY-non DDT efficacy)/2)

Units: Dmnl

proportion of population covered by non DDT IRS= Population Covered By Non DDT IRS Application/total population living in malaria risk areas

Units: Dmnl

SHARE OF EM EXPENDITURE FOR INFRASTRUCTURE AND HOUSEHOLD MODIFICATIONS TIME SERIES([(1970,0)-(2050,1.2)],(1970,1),(2005,1),(2050,0.5))

Units: Dmnl

SHARE of IRS budget for DDT

Units: Dmnl

TIME FOR EM IMPLEMENTATION= 3

Units: Year

TIME FOR IRS DELPOYMENT= 1

Units: Year

TIME FOR NON BEDNET PROTECTIVE MEASURES IMPLEMENTATION= 1

Units: Year

total area covered by environmental management= Area Covered By EM+area covered by larviciding

Units: Square kilometer

total proportion of population covered by IRS= population effectively covered by DDT+population effectively covered by non DDT IRS

Units: Dmnl

Treating Areas With EM= DELAY N(EM expenditure for infrastructure and household modifications/EM UNIT COST, TIME FOR EM IMPLEMENTATION, 0.555, 1)

Units: Square kilometer/Year

********************************

8 Malaria treatment

********************************

effect of education level on prompt access to formal health care= EFFECT OF EDUCATION LEVEL ON PROMPT ACCESS TO FORMAL HEALTH CARE TABLE(relative average years schooling)

Units: Dmnl

EFFECT OF EDUCATION LEVEL ON PROMPT ACCESS TO FORMAL HEALTH CARE TABLE([(0,0)-(10,1)],(0,0.2),(9,1))

Units: Dmnl

EFFECT OF EFFICIENTLY COVERED POPULATION ON MALARIA DEATH RATE( [(0,0)-(1,1)],(0,1),(1,0.1))

Units: Dmnl

EFFECT OF HEALTH EXPENDITURE ON HEALTH SERVICES COVERAGE([(0,0)-(100,1)],(10,0.4),(20,0.5),(30,0.58),(40,0.65),(100,1))

Units: Dmnl

effect of pc malaria treatment expenditure on access to treatment= EFFECT OF PC MALARIA TREATMENT EXPENDITURE ON ACCESS TO TREATMENT TABLE(Malaria Treatment Expenditure Per Malaria Case)

Units: Dmnl

EFFECT OF PC MALARIA TREATMENT EXPENDITURE ON ACCESS TO TREATMENT TABLE([(0,0)-(4,1)],(0,0.2),(4,1))

Units: Dmnl

effect of treatment coverage on malaria mortality= EFFECT OF EFFICIENTLY COVERED POPULATION ON MALARIA DEATH RATE(percentage of infected people attended in formal health care services)

Units: Dmnl

EFFECTIVE HEALTH EXPENDITURE ADJUSTMENT TIME= 5

Units: Year

Effective Pc Health Expenditure= SMOOTH N(pc health expenditure, EFFECTIVE HEALTH EXPENDITURE ADJUSTMENT TIME, 21.5, 1)

Units: $/(Person*Year)

effective total pc health expenditure= Effective Pc Health Expenditure

Units: $/(Year*Person)

health services coverage= EFFECT OF HEALTH EXPENDITURE ON HEALTH SERVICES COVERAGE(effective total pc health expenditure)

Units: Dmnl

malaria high quality therapies access= health services coverage*effect of pc malaria treatment expenditure on access to treatment

Units: Dmnl

Malaria Treatment Expenditure Per Malaria Case= SMOOTH N(IF THEN ELSE(total estimated malaria cases>10, malaria treatment expenditure/total estimated malaria cases, 10), 1, 0.26, 1)

Units: $/Person

percentage of infected people attended in formal health care services= malaria high quality therapies access*effect of education level on prompt access to formal health care

Units: Dmnl

relative average years schooling= Average Years Of Schooling/INITIAL AVERAGE YEARS OF SCHOOLING

Units: Dmnl

********************************

9 Malaria cost accounting

********************************

asymptomatic infected population= SUM(Malaria Infectious And Partially Immune Population[sex!,age!])*ASYMPTOMATIC POPULATION FRACTION

Units: Person

ASYMPTOMATIC POPULATION FRACTION= 0.6

Units: Dmnl

AVERAGE SALARY ADJUSTMENT FACTOR= 5

Units: Dmnl

average salary in IVM sector= theoretical average salary*AVERAGE SALARY ADJUSTMENT FACTOR

Units: $/(Year*Person)

bednet distribution employment= bednets distributed*EMPLOYMENT PER BEDNET DISTRIBUTED

Units: Person

bednet expenditure= integrated vector management interventions budget*normalized share of IVM budget for bednet

Units: $/Year

bednet production and distribution employment= bednet production employment+bednet distribution employment

Units: Person

bednet production employment= bednets distributed*EMPLOYMENT PER BEDNET PRODUCED

Units: Person

bednets distributed= budget for bednets/BEDNET UNIT COST

Units: Bednet/Year

CAPITAL SHARE= 0.35

Units: Dmnl

DDT On Dwellings= INTEG (DDT application rate to dwellings-degradation rate on dwellings-evaporation rate from dwelling, 83402)

Units: Ton

effect of DDT on life expectancy= Effect Of DDT On Life Expectancy For Exposed Population*proportion of population covered by DDT

Units: Year

Effect Of DDT On Life Expectancy For Exposed Population= SMOOTH N(indicated effect of DDT on life expectancy, TIME FOR EFFECT OF DDT ON LIFE EXPECTANCY TO APPEAR, indicated effect of DDT on life expectancy, 1)

Units: Year

effect of malaria prevalence on productivity= 1-(productivity lost because of malaria-INITIAL PRODUCTIVITY LOST BECAUSE OF MALARIA)

Units: Dmnl

EM expenditure= integrated vector management interventions budget*normalized share of IVM budget for EM

Units: $/Year

EMPLOYMENT PER BEDNET DISTRIBUTED= 0.00014

Units: (Person*Year)/Bednet

EMPLOYMENT PER BEDNET PRODUCED= 0.00028

Units: (Person*Year)/Bednet

environmental management employement= (EM expenditure*FRACTION OF EM EXPENDITURE FOR SALARIES)/average salary in IVM sector

Units: Person

FRACTION OF EM EXPENDITURE FOR SALARIES= 0.5

Units: Dmnl

FRACTION OF NON DDT IRS EXPENDITURE FOR SALARIES= 0.5

Units: Dmnl

indicated effect of DDT on life expectancy= per capita DDT exposure in kg*YEARS OF LIFE EXPECTANCY LOST PER KG OF EXPOSURE

Units: Year

INITIAL PRODUCTIVITY LOST BECAUSE OF MALARIA= INITIAL(productivity lost because of malaria)

Units: Dmnl

integrated vector management interventions budget= malaria prevention expenditure

Units: $/Year

IRS expenditure= integrated vector management interventions budget*normalized share of IVM budget for IRS

Units: $/Year

KG PER TON= 1000

Units: Kg/Ton

labor force= SUM(Population[sex!, working age!])

Units: Person

MALARIA PAST PREVENTION EXPENDITURE TABLE([(1970,0)-(2050,2e+009)],(1970,3.74e+007),(1971,3.86e+007),(1972,3.95e+007),(1973,4.06e+007),(1974,4.17e+007),(1975,4.28e+007),(1976,4.39e+007),(1977,4.53e+007),(1978,4.67e+007),(1979,4.78e+007),(1980,4.91e+007),(1981,5.02e+007),(1982,5.14e+007),(1983,5.35e+007),(1984,5.45e+007),(1985,5.59e+007),(1986,5.75e+007),(1987,5.9e+007),(1988,6.1e+007),(1989,6.25e+007),(1990,6.45e+007),(1991,6.61e+007),(1992,6.81e+007),(1993,7.01e+007),(1994,7.21e+007),(1995,7.41e+007),(1996,7.61e+007),(1997,7.86e+007),(1998,8.11e+007),(1999,8.37e+007),(2000,8.62e+007),(2001,8.87e+007),(2002,9.14e+007),(2003,1.22e+008),(2004,2.02e+008),(2005,2.73e+008),(2006,4.36e+008),(2007,3.26e+008),(2008,4.95e+008),(2009,5.13e+008),(2010,5.29e+008),(2011,4.99e+008))

Units: $/Year

malaria prevention expenditure= IF THEN ELSE(Time<2011, MALARIA PAST PREVENTION EXPENDITURE TABLE(Time), MALARIA FUTURE PREVENTION EXPENDITURE TABLE(Time))

Units: $/Year

malaria treatment expenditure= IF THEN ELSE(Time<2011, MALARIA TREATMENT EXPENDITURE TABLE(Time),Perceived Total Estimated Malaria Cases*TARGET MALARIA TREATMENT COVERAGE(Time)*MALARIA TREATMENT UNIT COST)

Units: $/Year

MALARIA TREATMENT EXPENDITURE TABLE([(1970,0)-(2050,1e+009)],(1970,4.57e+007),(1971,4.71e+007),(1972,4.83e+007),(1973,4.97e+007),(1974,5.1e+007),(1975,5.23e+007),(1976,5.37e+007),(1977,5.53e+007),(1978,5.7e+007),(1979,5.85e+007),(1980,6e+007),(1981,6.14e+007),(1982,6.29e+007),(1983,6.53e+007),(1984,6.66e+007),(1985,6.84e+007),(1986,7.02e+007),(1987,7.21e+007),(1988,7.45e+007),(1989,7.64e+007),(1990,7.89e+007),(1991,8.07e+007),(1992,8.32e+007),(1993,8.56e+007),(1994,8.81e+007),(1995,9.06e+007),(1996,9.31e+007),(1997,9.61e+007),(1998,9.92e+007),(1999,1.02e+008),(2000,1.05e+008),(2001,1.08e+008),(2002,1.12e+008),(2003,1.5e+008),(2004,1.36e+008),(2005,2.27e+008),(2006,3.8e+008),(2007,5.44e+008),(2008,7.76e+008),(2009,8.04e+008),(2010,8.28e+008),(2011,8.67e+008))

Units: $/Year

maximum DDT coverage=SAMPLE IF TRUE(Population Covered By DDT Application>maximum DDT coverage, Population Covered By DDT Application, Population Covered By DDT Application)

Units: Person

non DDT IRS employment= non DDT IRS expenditure*FRACTION OF NON DDT IRS EXPENDITURE FOR SALARIES/average salary in IVM sector

Units: Person

non DDT IRS expenditure= IRS expenditure*fraction of chemical control expenditure for non DDT IRS

Units: $/Year

normalized share of IVM budget for bednet= IF THEN ELSE(Time<2012,SHARE of IVM budget for bednet,FUTURE SHARE OF IVM BUDGET FOR BEDNET(Time))/total percentage of budget requested

Units: Dmnl

normalized share of IVM budget for EM= IF THEN ELSE(Time<2012, SHARE of IVM budget for EM, FUTURE SHARE OF IVM BUDGET FOR EM(Time))/total percentage of budget requested

Units: Dmnl

normalized share of IVM budget for IRS= IF THEN ELSE(Time<2012,SHARE of IVM budget for IRS,FUTURE SHARE OF IVM BUDGET FOR IRS(Time))/total percentage of budget requested

Units: Dmnl

per capita DDT exposure in kg= DDT On Dwellings/maximum DDT coverage*KG PER TON

Units: Kg/Person

Perceived Total Estimated Malaria Cases= SMOOTH N(total estimated malaria cases, 1, 1.57e+008, 1)

Units: Person/Year

population affected by malaria symptoms= SUM(Malaria Infectious And Partially Immune Population[sex!,age!])-asymptomatic infected population

Units: Person

Population Covered By DDT Application= DELAY N(DDT expenditure/DDT UNIT COST,TIME FOR IRS DELPOYMENT,1.5e+007,1)

Units: Person

population fraction affected by malaria symptoms= MAX(0,population affected by malaria symptoms/total population)

Units: Dmnl

productivity lost because of malaria= population fraction affected by malaria symptoms*WORKING TIME LOSS BECAUSE OF MALARIA

Units: Dmnl

proportion of population covered by DDT= Population Covered By DDT Application/total population living in malaria risk areas

Units: Dmnl

real GDP= INITIAL PRODUCTION*relative production

Units: $/Year

SHARE of IVM budget for bednet

Units: Dmnl

SHARE of IVM budget for EM

Units: Dmnl

SHARE of IVM budget for IRS

Units: Dmnl

share of total malaria funding for prevention= malaria prevention expenditure/total funding for malaria control

Units: Dmnl

theoretical average salary= (real GDP*CAPITAL SHARE)/labor force

Units: $/(Year*Person)

TIME FOR EFFECT OF DDT ON LIFE EXPECTANCY TO APPEAR= 10

Units: Year

total estimated malaria cases= SUM(malaria cases[sex!,age!])

Units: Person/Year

total funding for malaria control= malaria treatment expenditure+malaria prevention expenditure

Units: $/Year

total percentage of budget requested= IF THEN ELSE(Time<2012, SHARE of IVM budget for IRS+SHARE of IVM budget for EM+SHARE of IVM budget for bednet, FUTURE SHARE OF IVM BUDGET FOR BEDNET(Time)+FUTURE SHARE OF IVM BUDGET FOR EM(Time)+FUTURE SHARE OF IVM BUDGET FOR IRS(Time))

Units: Dmnl

WORKING TIME LOSS BECAUSE OF MALARIA= 0.04

Units: Dmnl

YEARS OF LIFE EXPECTANCY LOST PER KG OF EXPOSURE= 1

Units: Year/(Kg/Person)

********************************

10 Indicators

********************************

Malaria Infectious And Partially Immune Population[sex,age]= INTEG (malaria cases[sex,age]-deaths due to other causes[sex,age]-infected recovered[sex,age]-Malaria Deaths[sex,age],Population[sex,age]*INITIAL MALARIA INFECTIOUS AND PARTIALLY IMMUNE FRACTION)

Units: Person

Population[sex,AGE 0]= INTEG (births[sex]-aging[sex,AGE 0]-deaths[sex,AGE 0], INITIAL population[sex, AGE 0])

Population[sex,all but youngest]= INTEG (migration[sex,all but youngest]-deaths[sex,all but youngest]+aging[sex,all but eldest]-aging[sex,all but youngest],INITIAL population[sex,all but youngest])

Units: Person

population by region[sex, age, region]= Population[sex,age]*PROPORTION of population by region[region]

Units: Person

population fraction affected by malaria= MAX(0,SUM(Malaria Infectious And Partially Immune Population[sex!,age!])/total population)

Units: Dmnl

population living in malaria risk areas[sex,age]= SUM(population living in malaria risk areas by region[sex,age,region!])

Units: Person

total population= SUM(Population[sex!,age!])

Units: Person

total population by region[region]= SUM(population by region[sex!,age!,region])

Units: Person

total population living in malaria risk areas= SUM(population living in malaria risk areas[sex!,age!])

Units: Person

total vulnerable population= SUM(vulnerable population[sex!,age!])

Units: Person

vulnerable population[sex,age]= MAX(1,population living in malaria risk areas[sex,age]*(1-integrated vector management coverage fraction))

Units: Person

vulnerable population fraction= total vulnerable population/SUM(Population[sex!,age!])

Units: Dmnl

********************************

11 Policy variables

********************************

AVERAGE BEDNET EFFECTIVE DURATION= 5

Units: Year

AVERAGE DURATION OF EFFECTIVENESS OF EM INTERVENTIONS= 8

Units: Year

BEDNET UNIT COST= 6

Units: $/Bednet

COVERAGE PER BEDNET= 2

Units: Person/Bednet

DDT UNIT COST= 4

Units: $/(Person*Year)

EM UNIT COST= 3000

Units: $/Square kilometer

FUTURE SHARE OF IRS BUDGET FOR DDT= 0.847

Units: Dmnl

FUTURE SHARE OF IVM BUDGET FOR BEDNET([(2011,0)-(2050,1)],(2012,0.55688),(2050,0.55))

Units: Dmnl

FUTURE SHARE OF IVM BUDGET FOR EM([(2011,0)-(2050,0.0001)],(2012,4.82544e-005),(2050,0.005))

Units: Dmnl

FUTURE SHARE OF IVM BUDGET FOR IRS([(2011,0)-(2050,1)],(2012,0.443072),(2050,0.445))

Units: Dmnl

INTERVENTIONS OVERLAPPING FACTOR= 0.5

Units: Dmnl

LARVICIDING UNIT COST= 1500

Units: $/Square kilometer/Year

MALARIA FUTURE PREVENTION EXPENDITURE TABLE([(1970,0)-(2050,3e+009)],(2011,5.45e+008),(2012,5.48e+008),(2013,5.88e+008),(2014,6.23e+008),(2015,6.54e+008),(2016,6.83e+008),(2017,7.1e+008),(2018,7.36e+008),(2019,7.62e+008),(2020,7.88e+008),(2021,8.13e+008),(2022,8.37e+008),(2023,8.62e+008),(2024,8.86e+008),(2025,9.11e+008),(2026,9.27e+008),(2027,9.43e+008),(2028,9.59e+008),(2029,9.76e+008),(2030,9.92e+008),(2031,1.01e+009),(2032,1.02e+009),(2033,1.04e+009),(2034,1.06e+009),(2035,1.08e+009),(2036,1.09e+009),(2037,1.11e+009),(2038,1.14e+009),(2039,1.16e+009),(2040,1.18e+009),(2041,1.21e+009),(2042,1.24e+009),(2043,1.27e+009),(2044,1.31e+009),(2045,1.35e+009),(2046,1.39e+009),(2047,1.43e+009),(2048,1.48e+009),(2049,1.53e+009),(2050,1.59e+009))

Units: $/Year

MALARIA TREATMENT UNIT COST= 4

Units: $/Person

MAX DDT EFFICACY= 0.6

Units: Dmnl

MAX NON DDT EFFICACY= 1

Units: Dmnl

NON DDT IRS UNIT COST= 6

Units: $/(Year*Person)

PROPORTIONAL REDUCTION IN RISK FOR EM COVERED POPULATION= 0.35

Units: Dmnl

PROPORTIONAL REDUCTION IN RISK FOR IRS COVERED POPULATION= 0.7

Units: Dmnl

TARGET MALARIA TREATMENT COVERAGE([(1970,0)-(2050,1)],(2010,0.6),(2025,0.5))

Units: Dmnl
